# Discovering the Elusive Global Minimum in a Ternary Chiral Cluster: Rotational Spectra of Propylene Oxide Trimer

**DOI:** 10.1002/anie.202010055

**Published:** 2020-10-07

**Authors:** Fan Xie, Marco Fusè, Arsh S. Hazrah, Wolfgang Jäger, Vincenzo Barone, Yunjie Xu

**Affiliations:** ^1^ Department of Chemistry University of Alberta Edmonton Alberta T6G 2G2 Canada; ^2^ Scuola Normale Superiore Piazza dei Cavalieri 7 56126 Pisa Italy

**Keywords:** conformational panorama, isotopic substitution, propylene oxide, rotational spectroscopy, semi-experimental equilibrium structures

## Abstract

The chirality controlled conformational landscape of the trimer of propylene oxide (PO), a prototypical chiral molecule, was investigated using rotational spectroscopy and a range of theoretical tools for conformational searches and for evaluating vibrational contributions to effective structures. Two sets of homochiral (PO)_3_ rotational transitions were assigned and the associated conformers identified with theoretical support. One set of heterochiral (PO)_3_ transitions was assigned, but no structures generated by one of the latest, advanced conformational search codes could account for them. With the aid of a Python program, the carbon atom backbone and then the heterochiral (PO)_3_ structure were generated using ^13^C isotopic data. Excellent agreement between theoretical and experimental rotational constants and relative dipole moment components of all three conformers was achieved, especially after applying vibrational corrections to the rotational constants.

## Graphical abstract

What can be done do when theory fails to provide a structural candidate? Using experimental isotopic data and a Python program, we establish a carbon backbone structure and eventually identify the elusive heterochiral propylene oxide trimer shown from 8^9^ initial candidates.

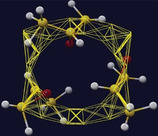

Propylene oxide (PO), a simple rigid chiral molecule, is widely used in the plastics industry as a building block for the synthesis of polyurethane polymers.^[1]^ PO has played an important role in the development of theoretical treatments of chiroptical spectroscopies, such as optical rotation dispersion and vibrational circular dichroism,^[2]^ and more recently in understanding solvent effects.^[3]^ PO is one of the earliest chiral molecules investigated in the microwave region,^[4]^ the foundation for its recent exciting discovery as the first chiral molecule in space.^[5]^ Effects of chirality recognition in several PO containing homo‐/heterochiral dimers^[6]^ were detected by using Fourier transform microwave (FTMW) spectroscopy where several conformers were identified experimentally. The advent of the chirped pulse (CP)‐FTMW^[7]^ technique and these early studies[^6^, ^8^] led to the development of chiral tag rotational spectroscopy for determination of chirality and enantiomeric excess (*ee*),^[9]^ a method which may potentially be applied to *ee* determination in space and contribute to our understanding of the origin of homochirality of life.^[10]^

So far, no high‐resolution spectroscopic study of PO aggregates beyond the dimer has been undertaken. Here we report the detection of multiple homo‐/heterochiral PO trimers using CP‐FTMW spectroscopy and point out unsolved problems in capturing the structural diversity of (PO)_3_ theoretically, even with advanced meta‐dynamic conformational search algorithms.^[11]^ We found that none of the structures generated in the theoretical conformational search can account for the experimental transitions of heterochiral (PO)_3_. By utilizing the classical isotopic substitution approach, we were able to establish the experimental heavy atom backbone of the heterochiral (PO)_3_, finally leading to the theoretical identification of its structure. Excellent agreement was achieved between experimental and predicted rotational constants using a general hybrid B2/B3^[12]^ and the further improved rPP/B3^[13]^ approach (vide infra) to account for vibrational contributions.

A pair of PO enantiomers, R and S, gives rise to two indistinguishable rotational spectra, whereas different homo‐/heterochiral diastereomeric PO aggregates can be formed (see Scheme 1).

**Scheme 1:**
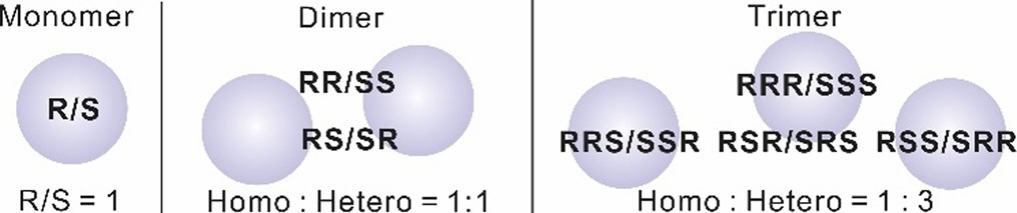
The statistical distribution of homo‐/heterochiral aggregates from monomer to trimer under racemic sample conditions, without consideration of the specific binding energies.

For (PO)_2_, specific binding topologies lead to 12 homo/ heterochiral conformers of which six were detected experimentally.^[6a]^ The much larger structural diversity of (PO)_3_ prompted us to employ the newly released CREST code,^[14]^ which has been successfully used for sampling conformational landscapes of organic molecules and their clusters.^[15]^ See Point S1, SI for structural search details. The resulting (PO)_3_ candidates were optimized at the B3LYP‐D3BJ^[16]^/def2‐TZVP level using Gaussian16.^[17]^ The true minimum structures identified within a small energy window of 5 kJ mol^−1^, which include 26 homochiral and 53 heterochiral (PO)_3_, are summarized in Tables S1,S2, SI. The large number of possible conformers within such a small energy window highlights the difficulties in identifying them experimentally.

Broadband spectra of homo‐/heterochiral (PO)_3_ were collected using a 2–6 GHz CP‐FTMW spectrometer.^[18]^ Experimental and spectral fitting details are summarized in Point S1, SI. In the enantiopure PO spectrum, transitions belonging to two homochiral (PO)_3_ conformers were assigned. A racemic PO spectrum is shown in Figure 1 where the two identified homochiral (PO)_3_ are clearly visible, as well as a new set of strong transitions. This new set was tentatively assigned to a heterochiral (PO)_3_ since the transitions are only visible with the racemic but not the enantiopure sample. The experimental rotational constants and relative magnitude of dipole moment components are compared with the DFT predictions in Table 1; the spectroscopic constants and the measured transition frequencies are listed in Tables S3–S6, SI. The spectroscopic properties of the two homochiral (PO)_3_ match those of the two most stable DFT homochiral (PO)_3_, HOMO1 and HOMO2, quite well, confirming their identification. No reasonable match to a DFT structure, however, could be made for the experimental heterochiral (PO)_3_ from the list of 53 heterochiral (PO)_3_ candidates (Table S2). For convenience, we name this experimental structure HETERO1.

**Figure 1:**
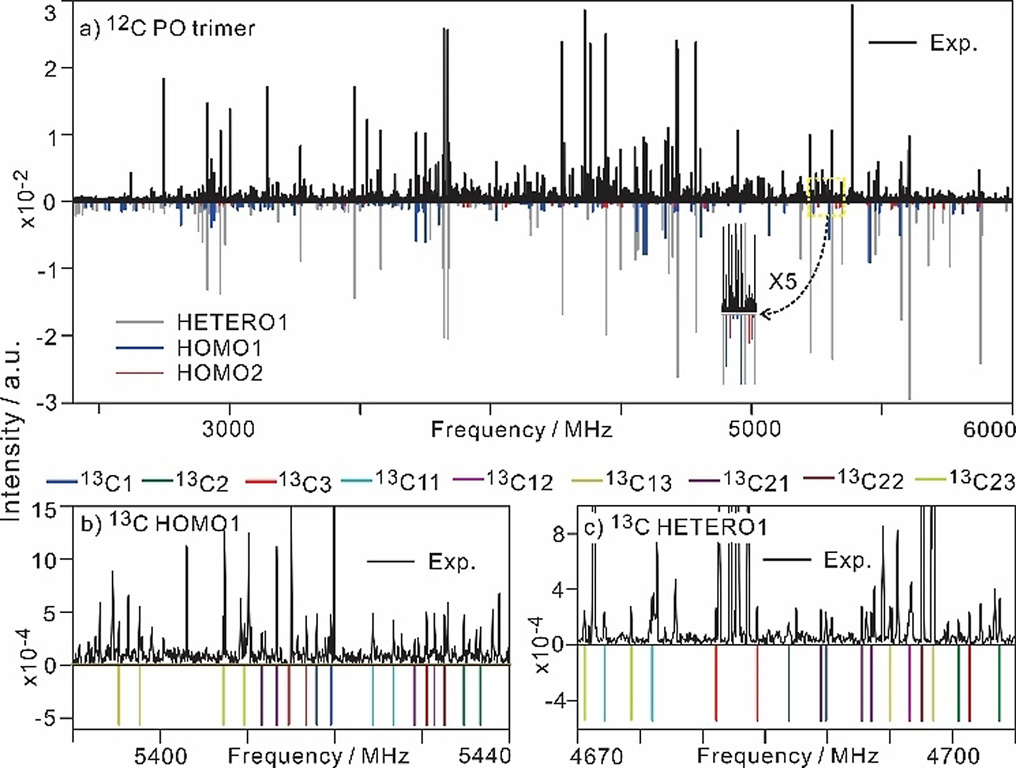
a) A broadband spectrum of (PO)_3_ recorded with 10^6^ FIDs using a racemic sample. Known lines of PO,^[4]^ PO‐Ne,^[19]^ (PO)_2_,^[6a]^ and PO‐(H_2_O)_1,2_[^3b^, ^3c^] were removed. The simulated spectra were generated with a rotational temperature of 1 K, experimental spectroscopic constants, calculated dipole moment components, and experimental relative abundance ratios of the three conformers. Bottom expanded sections recorded with b) enantiopure PO and 5×10^6^ FIDs, showing the JK_a_K_c_=606‐515 and 616‐505 transitions of ^13^C isotopologues of HOMO1 and c) a racemic PO sample and 6×10^6^ FIDs, showing the JK_a_K_c_=515‐414 and 505‐404 transitions of ^13^C isotopologues of HETERO1.

**Table 1:** Experimental and theoretical rotational constants (in MHz) and electric dipole moment components (in Debye) of the observed (PO)_3_ conformers.

Const.	HOMO1				HOMO2				HETERO1			
	exp.	B3LYP^[a]^	B2/B3^[b]^	rPP/B3^[b]^	exp.	B3LYP^[a]^	B2/B3^[b]^	rPP/B3^[b]^	exp.	B3LYP^[a]^	B2/B3^[b]^	rPP/B3^[b]^
*A*	836.79877(27)	829	829	835	782.7474(26)	791	783	784	811.72697(32)	800	801	810
*B*	638.95770(23)	648	639	638	628.8592(13)	644	631	629	645.92410(23)	656	646	645
*C*	430.04824(16)	433	427	431	458.53594(94)	471	460	457	444.53305(21)	448	443	443
												
*Dipole comp*.	*μ_b_*>*μ_a_*≈*μ_c_* ^[c]^	0.7/1.6 /0.8	0.8/1.5 /0.8	0.8/1.5 /0.9	*μ_a_*≈*μ_c_*, no *μ_b_* ^[c]^	1.1/0.2 /1.3	1.1/0.3 /1.4	1.1/0.3 /1.4	*μ_a_*>*μ_b_*>*μ_c_* ^[c]^	1.5/1.1 /0.7	1.6/1.0 /0.8	1.6/0.9 /0.8
												
*N* ^d^, *σ* ^[d]^	58, 3.1.	–	–	–	18, 3.2	–	–	–	69, 2.9	–	–	–

[a] B3LYP‐D3BJ/def2‐TZVP level. [b] Corrected for harmonic and anharmonic contributions. See the text for the abbreviations. [c] Experimentally estimated relative magnitudes. [d] *N* is the number of transitions included and *σ* is the standard deviation of the fit.

The failure to produce a possible HETERO1 structure theoretically is a significant issue since conformational diversity is important in a wide range of chemistry and biochemistry subfields. While we could not capture the structural diversity of (PO)_3_ in all details in our theoretical conformational search, the subtly different binding topologies can be easily discriminated by the CP‐FTMW method with its very high spectral resolution. We therefore resorted to use isotopic substitution, a hallmark of rotational spectroscopy, to further explore the structural details of (PO)_3_ experimentally.

To map out the heavy atom backbone of (PO)_3_, it is ideal to have a single substitution at each heavy atom. Since the enriched ^13^C and ^18^O isotopologues of PO are prohibitively expensive, we optimized the experimental conditions and utilized the sensitivity achieved to measure and assign *all* 18 ^13^C isotopologues of HOMO1 and HETERO1 in their natural abundances. Figures 1 b and c) show some transitions of ^13^C isotopologues of HOMO1 and HETERO1; the experimental transition frequencies and spectroscopic constants are in Tables S7–S10, SI. The ^13^C isotopologues of HOMO2 could not be detected because of the too low abundance (see Figure 1).

The Kraitchman coordinates for singly substituted ^13^C atoms are summarized in Tables S11 and S12 of the SI for HOMO1 and HETERO1, respectively. Since Kraitchman's analyses provide only absolute values of the C atom coordinates, there are 8^9^ possible C‐backbone configurations without considering any chemical constraints because each of the nine carbon atoms has three Cartesian coordinates, leading to a total of (2^3^)^9^=8^9^ possibilities. For HOMO1, we used the DFT geometry as a guide to determine the signs. The experimental substitution structure of the carbon backbone frame of HOMO1 is compared with the final DFT one (vide infra) in Figure 2 a).

**Figure 2:**
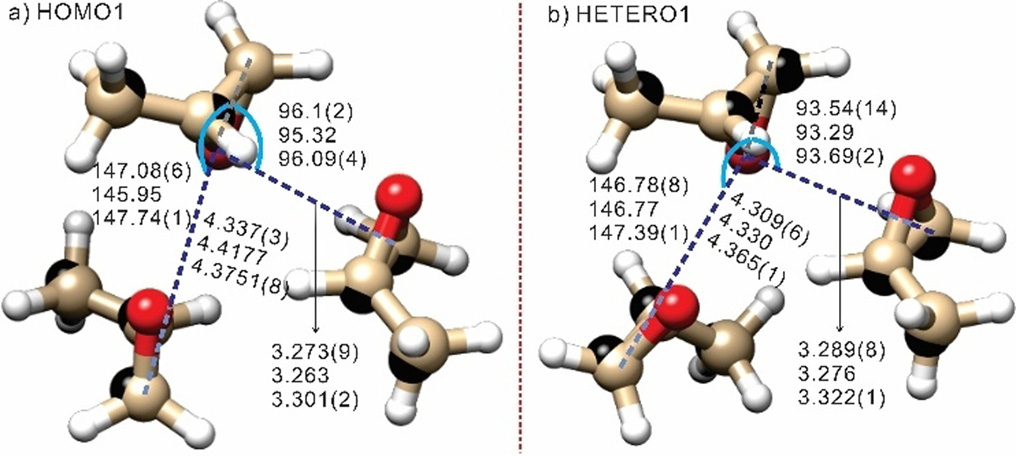
The rPP equilibrium structures of a) HOMO1 and b) HETERO1, together with the C‐backbones (black balls) based on Kraitchman coordinates. Some key intermolecular structural parameters (bond lengths in [Å] and angles in [°]) are listed in order of semi‐experimental equilibrium, rPP equilibrium, and experimental effective ones. Red: O‐atoms; white: H‐atoms; bone: C‐atoms.

For HETERO1, a Python program was written to extract one unique structural candidate out of the 8^9^ possibilities: by utilizing the known PO structure and setting limits for the intramolecular C−C distances, a few hundred possible C‐backbone candidates were obtained initially. The O and H atoms were then put back in, based on the PO monomer geometry. Configurations in which the PO subunits are too close or too far away were then removed, leading to just one unique (PO)_3_ structure. We tested this procedure first with HOMO1, and then applied it to HETERO1. Details of the search are given in Point S2, SI. The derived HETERO1 C‐backbone is shown in Figure 2 b), together with the ab initio structure based on it. We note that the calculated rotational constants and electric dipole moment components of this DFT HETERO1 structure match the experiment results in Table 1. Interestingly, HOMO1 is made of RR6 and RR3 PO dimer subunits, HOMO2 utilizes the somewhat distorted binding topologies of RR2 and RR4, and HETERO1 contains RR6 and RS3,^[6]^ whereas the third PO pair in these trimers utilizes mainly one CH⋅⋅⋅O contact for the interaction. (See Ref. [6] for notation of PO dimer units.)

The experimental relative abundance ratio of HETERO1:HOMO1:HOMO2 is estimated to be 3.3:1.0:0.2 for the racemic sample. If their stabilities were the same, one would expect the HETERO1 versus HOMO1 ratio to be 3, based on statistical arguments (Scheme 1). The abundance ratio indicates that the experimental stability decreases from HETERO1 (most stable) to HOMO1 and then to the much less stable HOMO2. While the experimental intensities of HOMO1 and HOMO2 are consistent with the B3LYP energy ordering in Table S1, HETERO1 is predicted to be ≈0.1 kJ mol^−1^ less stable than HOMO1 (Table S2).

In the above discussion, experimental vibrational ground‐state rotational constants (*B*_0_) are compared with the equilibrium values (*B*_e_). For fully quantitative purposes, the difference between them cannot be neglected. Here we applied a general hybrid approach, referred to as B2/B3, which is based on harmonic/anharmonic force fields computed with the B2PLYP/B3LYP‐D3BJ functionals in conjunction with jun‐cc‐pVTZ and jun‐cc‐pVDZ basis sets, respectively. The B2/B3 approach has been validated for both covalent interactions and non‐covalently bound complexes.^[12]^ In addition, we replaced the B2PLYP‐D3 functional by the revised version of the DSD‐PBEP86‐D3BJ functional (abbreviated as rPP),^[13]^ which further improves results for non‐covalent interactions over B2.

The six lowest homo‐/heterochiral structures, including HETERO1, were re‐optimized at the B2 and rPP levels and anharmonic vibrational corrections were computed at the B3 level. A full account of the computational details is given in Point S3, SI. The resulting *B*_e_ values, relative energies, dipole moment components at the B2 and rPP levels, and their vibrational corrections are summarized in Tables S13‐S18, SI. The results for all singly substituted ^13^C isotopologues of HOMO1, HOMO2, and HETERO1 are in Tables S19–S30, SI. It is noteworthy that at the rPP level, HETERO1 becomes the global minimum, thus restoring full agreement with the experimental energy ordering. The maximum difference between experimental rotational constants and those from the B2/B3 hybrid Scheme is *reduced* to 1.2 % in comparison to 3.7 % with just the equilibrium calculations, and the new hybrid rPP/B3 model further reduces the difference to below 0.3 % for all (PO)_3_ observed (Table 1). This remarkable level of accuracy also allowed us to confidently identify the HOMO2 carrier. With the vibrational corrections, we obtained the 20 sets of semi‐experimental equilibrium rotational constants (normal and ^13^C isotopologues) of HOMO1 and HETERO1, which were used to refine their theoretical equilibrium structures while fixing the monomer geometries and some intermolecular structural parameters. The refined results are given in Tables S31–34, SI, with some key parameters shown in Figure 2.

In conclusion, the conformational panorama of (PO)_3_, a ternary chiral system of fundamental interest, was investigated in detail using CP‐FTMW spectroscopy and a range of theoretical treatments including the CREST, B3LYP, B2/B3, and rPP/B3 calculations. The high sensitivity/resolution capability of the CP‐FTMW instrument allowed us to uncover the elusive DFT HETERO1 structure based on experimental ^13^C isotopic data. The study further reveals a slight chirality controlled conformational preference for the heterochiral trimer, continuing the same preference shown in (PO)_2_. The highly sophisticated semiempirical conformational search program CREST was not able to find the most stable ternary heterochiral aggregate, and the current work may provide benchmark data for further improvements.

## Conflict of interest

The authors declare no conflict of interest.

## Supporting information

As a service to our authors and readers, this journal provides supporting information supplied by the authors. Such materials are peer reviewed and may be re‐organized for online delivery, but are not copy‐edited or typeset. Technical support issues arising from supporting information (other than missing files) should be addressed to the authors.

Supplementary
