# Supplementary material for: Discovering the Elusive Global Minimum in a Ternary Chiral Cluster: Rotational Spectra of Propylene Oxide Trimer
Source: Angew Chem Int Ed Engl. 2020 Oct 7;59(50):22427–30. doi: 10.1002/anie.202010055 (PMC7984290; doi:10.1002/anie.202010055)
Supplement: Supplementary file 1 — Supplementary [file ANIE-59-22427-s001.pdf]

Angewandte Chemie
Eine Zeitschrift der Gesellschaft Deutscher Chemiker

Supporting Information

Discovering the Elusive Global Minimum in a Ternary Chiral Cluster: Rotational Spectra of Propylene Oxide Trimer

Fan Xie, Marco Fusè, Arsh S. Hazrah, Wolfgang Jäger, Vincenzo Barone, and Yunjie Xu**

anie_202010055_sm_miscellaneous_information.pdf

Supporting Information

Contents:

1. Table S1-S2. Energies and spectroscopic constants of the homo- and heterochiral $(PO)_3$ at the B3LYP-D3(BJ)/def2-TZVP level…………..…………………………………………..…………........…………S2-S3

2. Point S1 Experimental and spectral fitting details……………………………………………………………S4

3. Table S3 Experimental spectroscopic constants of the three $(PO)_3$ observed…..…………………,……S4

4. Tables S4-S6 Measured transition frequencies of the observed $(PO)_3$ conformers…………..……..S5-S8

5. Table S7-S10 Experimental spectroscopic constants of the 18 ^{13}C isotopologues of HOMO1 and HETERO1 and their transition frequencies…………..……………………………………………….. S9-S14

6. Table S11-S12. Kraitchman's coordinates of the C atoms in HOMO1 and HETERO1……..…..S15-S16

7. Point S2 Details of the Python program used for extracting the heavy atom frame…………....….......S17

8. Point S3 Details of the hybrid approach for evaluating harmonic and anharmonic contributions….…S18

9. Table S13-S30 Results of B2/B3 and rPP calculations for HOMO1 and HETERO1…………..….S19-S25

10. Table S31-S34 Equilibrium and semi-experimental equilibrium structural parameters of HOMO1 and HETERO1…………………………………………………………………………………….…...S26-S27

11. Completion of reference 16………………………………………………………………………………S28

Table S1. The theoretical raw (ΔD_e) and *ZPE* corrected (ΔD_0) relative dissociation energy (kJ mol^{-1}), rotational constants (MHz) and electric dipole moments (Debye) of homochiral PO trimers within a window of 5 kJ mol^{-1} at the B3LYP-D3BJ/def2-TZVP level of theory.

Conformer	ΔD_e	ΔD_0	A	B	C	$\|\mu_a\|$	$\|\mu_b\|$	$\|\mu_c\|$
**HOMO1**[a]	**0.8**	**0.0**	**829**	**648**	**434**	**0.7**	**1.6**	**0.8**
**HOMO2**[a]	**0.0**	**0.4**	**791**	**644**	**471**	**1.1**	**0.2**	**1.4**
HOMO3	1.2	1.1	784	654	422	0.9	1.1	1.0
HOMO4	1.9	1.2	763	655	445	1.0	0.2	1.5
HOMO5	1.9	1.2	795	618	449	0.1	1.2	0.1
HOMO6	2.0	1.2	758	669	394	1.5	0.1	1.0
HOMO7	2.4	2.1	926	553	422	1.1	0.1	1.5
HOMO8	3.4	2.6	861	538	400	0.9	0.1	0.8
HOMO9	3.6	2.8	812	551	384	2.1	0.6	0.3
HOMO10	3.2	2.9	939	562	420	1.0	0.1	1.2
HOMO11	4.1	2.9	680	680	448	0.0	0.0	0.7
HOMO12	4.2	3.0	761	587	368	2.3	0.7	0.4
HOMO13	4.1	3.5	1463	328	318	0.2	1.4	1.2
HOMO14	4.1	3.5	814	623	428	0.9	0.1	1.4
HOMO15	4.9	3.6	774	639	420	2.0	0.4	0.2
HOMO16	4.8	3.6	702	626	383	0.6	1.1	1.6
HOMO17	5.2	3.6	880	560	393	0.2	2.0	0.5
HOMO18	4.3	3.7	1382	335	302	0.1	1.7	0.8
HOMO19	4.5	3.7	1452	320	293	0.3	1.3	1.2
HOMO20	4.5	3.7	883	577	442	1.4	0.0	1.2
HOMO21	4.4	3.7	786	568	374	1.3	0.2	1.5
HOMO22	4.9	3.8	1549	330	299	0.5	1.2	1.6
HOMO23	5.8	4.3	926	548	407	0.1	1.6	0.3
HOMO24	5.8	4.4	916	550	407	0.1	1.6	0.4
HOMO25	6.1	4.4	905	500	378	0.5	1.2	0.9
HOMO26	5.9	5.0	1010	497	394	0.7	1.6	0.6

[a] The red color indicates the conformers detected experimentally.

Table S2. The theoretical raw (ΔD_e) and *ZPE* corrected (ΔD_0) relative dissociation energy (kJ mol^{-1}), rotational constants (MHz) and electric dipole moments (Debye) of homochiral PO trimers within a window of 5 kJ mol^{-1} at the B3LYP-D3BJ/def2-TZVP level of theory.[a]

Conformer	ΔD_e	ΔD_0	A	B	C	$\|\mu_a\|$	$\|\mu_b\|$	$\|\mu_c\|$
HETERO1o[b]	0.0	0.0	726	687	439	0.2	1.8	0.8
**HETERO1**[a,c]	**0.8**	**0.1**	**800**	**656**	**448**	**1.5**	**1.1**	**0.7**
HETERO2	0.3	0.1	744	672	457	1.3	0.1	1.1
HETERO3	0.7	0.6	807	604	463	1.4	0.4	1.1
HETRTO4	0.2	0.8	888	580	441	1.1	0.4	1.1
HETERO5	1.2	0.8	768	679	427	1.0	0.5	1.1
HETERO6	1.4	1.0	875	621	448	0.7	1.6	0.3
HETERO7	0.8	1.2	801	621	438	1.1	0.5	1.5
HETERO8	1.6	1.2	845	572	421	0.2	1.1	0.1
HETERO9	1.6	1.3	752	621	444	1.2	0.4	0.3
HETERO10	1.5	1.5	866	607	424	1.4	0.1	1.0
HETERO11	1.5	1.6	763	651	471	1.1	0.4	0.9
HETERO12	1.6	1.7	864	592	426	0.9	0.5	1.2
HETERO13	2.0	1.8	851	613	413	1.1	0.6	0.9

HETERO14	2.3	1.8	751	633	457	1.2	0.4	0.2
HETERO15	2.3	1.9	748	651	411	0.1	1.3	1.0
HETERO16	2.7	2.0	790	655	438	1.2	0.6	0.7
HETERO17	2.2	2.1	794	620	465	0.7	0.6	0.6
HETERO18	2.3	2.2	872	580	431	0.9	0.3	0.7
HETERO19	2.1	2.2	1104	468	395	0.8	1.4	0.7
HETERO20	2.4	2.3	763	647	430	1.0	0.0	1.5
HETERO21	2.3	2.4	795	616	425	1.3	0.4	1.1
HETERO22	2.8	2.5	875	545	406	0.5	0.8	1.1
HETERO23	2.3	2.5	686	657	413	1.2	0.7	1.4
HETERO24	3.0	2.6	893	585	426	0.7	0.7	1.5
HETERO25	3.1	2.7	776	638	456	0.5	1.2	0.3
HETERO26	2.9	2.7	840	563	416	1.1	0.0	0.9
HETERO27	3.6	2.8	694	669	447	0.0	0.4	0.1
HETERO28	3.1	2.9	731	612	387	1.2	0.0	1.2
HETERO29	3.5	2.9	793	556	399	1.5	0.0	0.1
HETERO30	3.2	3.0	986	539	416	0.2	1.0	0.6
HETERO31	3.8	3.1	807	631	425	0.8	0.4	1.1
HETERO32	3.9	3.1	839	608	444	2.6	0.6	0.8
HETERO33	3.5	3.2	1487	346	318	0.5	1.1	1.5
HETERO34	3.6	3.3	936	547	425	0.9	0.2	1.3
HETERO35	3.3	3.4	1320	368	329	0.2	1.1	1.5
HETERO36	3.9	3.4	871	580	465	1.5	0.1	1.2
HETERO37	3.8	3.5	1260	381	322	0.5	0.6	1.9
HETERO38	3.7	3.5	1541	325	314	0.8	1.9	0.4
HETERO39	4.1	3.6	1051	479	376	0.6	0.2	1.4
HETERO40	4.0	3.7	1058	429	348	0.6	1.2	1.3
HETERO41	4.2	3.8	783	607	403	2.2	0.8	0.6
HETERO42	4.0	3.8	1474	346	317	0.8	1.2	1.3
HETERO43	4.1	3.9	1219	377	313	0.1	1.3	1.4
HETERO44	4.5	3.9	1326	379	317	0.8	1.1	1.4
HETERO45	4.6	4.0	1059	470	380	1.2	0.6	1.2
HETERO46	4.7	4.0	1540	330	298	0.8	1.2	1.4
HETERO47	5.0	4.1	844	600	437	0.4	2.4	0.0
HETERO48	4.7	4.2	778	666	443	2.4	1.8	0.7
HETERO49	5.0	4.3	1528	335	307	0.2	1.5	0.9
HETERO50	5.6	4.3	883	566	421	0.3	2.3	0.0
HETERO51	4.9	4.4	800	609	447	2.6	1.3	1.1
HETERO52	5.3	4.5	1312	385	353	0.4	1.6	0.9
HETERO53	5.1	4.8	759	670	434	2.1	1.3	0.6

[a] All conformers are sampled from the CREST search *except HETERO1* which was ONLY identified using the isotopic procedure described in the main text.

[b] ΔD_0 of HETERO1o is 0.1 kJ mol^{-1} **less** stable than HOMO1, inconsistent with the experimental observation. See main text for discussions.

[c] The red color indicates the conformer detected experimentally.

Point S1. Structural searching and experimental and spectral fitting details

For the conformational structural search of the homo- and heterochiral PO trimers, we applied the most recent version of CREST (version 2.6) [S1]. Each CREST run contains multiple runs of META MD and then multiple runs of regular MD as designed by the code [S1]. As reported by the authors of this version of CREST, the search results do not depend on the starting structures. We have identified the homochiral species in agreement with the experimental observation as discussed in the main text. For the heterochiral PO trimer, we carried out multiple CREST runs with different starting structures, including the HETERO1 structure identified experimentally, but CREST did not produce the correct HETERO1 structure in all these runs. The HETERO1 structure was identified using isotopic substitution data as described in the main text.

For measurements of the broadband spectra of the PO sample, a modest MW power set at ~100 W, about ¼ of the full power available, was used. Enantiomeric pure (>99.0%) and racemic PO (99%) samples from Sigma Aldrich were used as is. The liquid PO samples were treated with the usual freeze-thaw cycles and a gas mixture of less than 1% PO in 9 bar helium and 1 bar neon was expanded through a home-made nozzle cap with an exit channel length of 25 mm and an exit hole diameter of 1 mm which was described in a recent publication [S2]. 1 million to 6 million free induction decays were collected for spectral analyses of the parents and the ^{13}C isotopologues.

Two sets of rotational transitions belonging to the homochiral $(PO)_3$ conformers were assigned in the spectrum recorded using an enantiomerically pure PO sample. One set of rotational transitions belonging to the heterochiral PO trimer were assigned in the spectrum recorded using a racemic PO sample and are not visible in the spectrum recorded using the enantiomerically pure PO sample.

The three sets of rotational transitions of $(PO)_3$, as well as 18 sets of ^{13}C isotopologues of $(PO)_3$ were fitted using Watson's A-reduction [S3] Hamiltonian in its I^r representation with the Pgopher program [S4].

[S1] S. Grimme, C. Bannwarth, P. Shushkov, *J. Chem. Theory Comput.* **2017**, *13*, 1989-2009; P. Pracht, F. Bohle, S. Grimme, *Phys. Chem. Chem. Phys.* **2020**, 22, 7169-7192.

[S2] F. Xie, N. A. Seifert, W. Jaeger, Y. Xu, *Angew. Chem. Int. Ed.* **2020**, DOI:10.1002/anie.202005685 and 10.1002 /ange.202005685.

[S3] J. K. G. Watson, in *Vibrational Spectra and Structure*, Vol. 6 (Ed.: J. R. Durig), Elsevier, New York, **1977**, pp. 1- 89.

[S3] C. M. Western, *J. Quant. Spectrosc. Radiat. Transf.* **2017**, *186*, 221-242.

Table S3. Experimental spectroscopic constants of the three observed $(PO)_3$.

	HOMO1	HOMO2	HETERO1
A / MHz	836.79877(27)	782.7474(26)	811.72697(32)
B / MHz	638.95770(23)	628.8592(13)	645.92410(23)
C / MHz	430.04824(16)	458.53594(94)	444.53305(21)
Δ_K / kHz	-0.004(13)	0.41(21)	0.167(21)
Δ_{JK} / kHz	0.297(11)	0.252(90)	0.012(22)
Δ_J / kHz	0.1658(26)	0.152(28)	0.1886(50)
δ_K / kHz	0.2850(74)	-0.062(58)	0.098(14)
δ_J / kHz	0.0497(14)	0.037(12)	0.0550(22)
N^a	58	18	69
σ^a / kHz	3.1	3.2	2.9

[a] N is the number of transitions included and σ is the standard deviation of the fit.

Table S4. Experimental transition frequencies of HOMO1.

J'	K_a'	K_c'	J''	K_a''	K_c''	ν_{EXP} / MHz	$\Delta\nu^a$/ MHz
6	0	6	5	1	5	5451.4210	-0.0037
6	1	6	5	0	5	5453.5070	0.0001
5	1	5	4	0	4	4597.2300	0.0043
5	0	5	4	1	4	4588.1980	0.0044
4	0	4	3	1	3	3715.4270	-0.0014
4	1	4	3	0	3	3750.9360	0.0005
4	3	2	3	2	1	5570.2292	-0.0061
6	2	5	5	1	4	6080.5490	-0.0005
7	0	7	6	1	6	6312.1545	0.0095
7	1	7	6	0	6	6312.5879	-0.0085
5	4	2	5	3	3	2098.6262	0.0075
2	1	2	1	0	1	2126.9497	0.0098
5	3	3	5	2	4	2128.7940	-0.001
6	4	3	6	3	4	2303.8516	-0.0016
6	2	4	6	1	5	2358.5762	-0.0025
5	5	1	5	4	2	2524.2660	-0.0022
6	5	2	6	4	3	2524.4610	0.0001
5	1	4	5	0	5	2536.8257	0.0022
6	3	4	6	2	5	2586.6403	-0.0016
5	2	4	5	1	5	2594.6895	0.0012
7	5	3	7	4	4	2602.1686	0.0004
7	4	4	7	3	5	2641.1001	0.0029
8	6	2	8	5	3	2643.6583	0.0011
8	5	4	8	4	5	2799.6631	-0.0031
8	3	5	8	2	6	2805.4742	0.0018
3	0	3	2	1	2	2812.6278	-0.0032
7	6	1	7	5	2	2909.7246	-0.0002
3	1	3	2	0	2	2929.7524	-0.0011
2	2	1	1	1	0	2940.4320	-0.0015
3	1	3	2	1	2	2840.8149	0.0001
3	0	3	2	0	2	2901.5696	0.0001
3	2	2	2	2	1	3206.9928	0.0002
3	1	2	2	1	1	3433.2488	-0.0001
3	2	1	2	2	0	3512.4230	-0.0001
4	1	4	3	1	3	3722.7516	-0.0001
4	0	4	3	0	3	3743.6123	0.0001
4	2	3	3	2	2	4193.6343	-0.0001
4	1	3	3	1	2	4391.1516	-0.0001
4	3	2	3	3	1	4411.3833	0.0001
4	3	1	3	3	0	4578.5912	-0.0001
5	0	5	4	0	4	4595.5168	-0.0001
5	1	5	4	1	4	4589.9026	-0.0002
4	2	2	3	2	1	4716.3634	0.0002

5	2	4	4	2	3	5127.2875	-0.0001
2	1	1	1	0	1	2753.6613	-0.0001
2	2	0	1	1	0	3040.4389	-0.0002
2	2	1	1	1	1	3149.3415	-0.0001
3	1	2	2	0	2	4148.9089	0.0001
3	2	1	2	1	1	4205.9498	-0.0001
3	2	2	2	1	2	4427.2356	0.0002
3	3	0	2	2	0	4705.5553	-0.0001
3	3	1	2	2	1	4771.2804	-0.0001
4	2	2	3	1	2	5489.0644	0.0001
4	1	3	3	0	3	5638.4908	0.0001
6	2	4	5	3	2	5637.3569	-0.0001
4	3	1	3	2	1	5771.7235	0.0001
4	2	3	3	1	3	5780.0550	0.0001
4	3	2	3	2	2	5975.6709	-0.0001

[a] $\nu_{obs.} - \nu_{cacl}$.

Table S5. Experimental transition frequencies of HOMO2.

J'	K_a'	K_c'	J''	K_a''	K_c''	ν_{EXP} / MHz	$\Delta\nu^a$ / MHz
6	0	6	5	0	5	5738.1170	-0.0043
6	1	6	5	1	5	5737.2030	0.0010
5	1	5	4	1	4	4818.8700	0.0091
5	0	5	4	0	4	4822.8600	-0.0029
4	3	2	3	2	2	5701.1820	-0.0006
4	2	3	3	1	3	5552.5950	0.0006
5	3	3	4	3	2	5541.8635	-0.0003
4	3	1	3	2	1	5536.9507	0.0024
5	2	4	4	2	3	5254.9358	0.0013
4	2	2	3	1	2	5325.8739	0.0006
5	1	4	4	1	3	5337.8080	0.0009
4	1	3	3	0	3	5447.9720	0.0009
4	2	2	3	2	1	4709.3618	0.0013
4	3	1	3	3	0	4604.5873	-0.0020
4	1	3	3	1	2	4435.1041	-0.0070
3	3	0	2	2	0	4447.4231	-0.0028

[a] $\Delta\nu = \nu_{exp} - \nu_{cacl}$.

Table S6. Experimental transition frequencies of HETERO1.

J'	K_a'	K_c'	J''	K_a''	K_c''	ν_{EXP} / MHz	$\Delta\nu^a$ / MHz
6	0	6	5	1	5	5606.0070	-0.0025
6	1	6	5	1	5	5606.2001	0.0007
6	0	6	5	0	5	5606.9710	-0.0011
6	1	6	5	0	5	5607.1631	0.0011
5	0	5	4	1	4	4715.0177	-0.0012
5	1	5	4	1	4	4715.9791	-0.0024
5	0	5	4	0	4	4719.5900	-0.0011
5	1	5	4	0	4	4720.5520	-0.0017
5	3	3	4	3	2	5575.3112	0.0017
5	4	2	4	4	1	5677.7210	0.0029
5	4	1	4	4	0	5760.1747	-0.0054
5	2	3	4	2	2	5876.8008	0.0001
5	3	2	4	3	1	5985.1505	0.0026
6	1	5	5	2	4	6131.4808	-0.0045
6	2	5	5	2	4	6141.4410	-0.001
6	1	5	5	1	4	6169.4353	-0.0031
6	2	5	5	1	4	6179.4001	0.005
4	4	1	3	3	0	6219.8824	-0.0015
5	3	3	4	2	2	6261.4125	-0.0032
4	4	0	3	3	1	6271.3755	0.0056
6	3	4	5	3	3	6590.6761	-0.0009
6	2	4	5	2	3	6812.6709	0.004
6	4	3	5	4	2	6813.6440	0.0075
2	2	1	1	1	0	2879.7108	0.0052
3	0	3	2	1	2	2895.2364	-0.0061
3	1	3	2	1	2	2914.7710	-0.001
7	3	5	7	2	6	2918.3626	-0.0068
7	3	5	7	1	6	2920.7135	0.0002
3	0	3	2	0	2	2963.6500	-0.0015
3	1	3	2	0	2	2983.1893	0.0083
2	2	0	1	1	1	3185.0864	-0.0043
3	2	2	2	2	1	3271.3515	0.0007
3	1	2	2	1	1	3479.6443	0.0016
3	2	1	2	2	0	3579.0494	-0.0009
3	2	2	2	1	1	3768.7588	-0.0004
4	0	4	3	1	3	3817.1616	0.0005
4	1	4	3	1	3	3821.7338	0.0005
4	0	4	3	0	3	3836.6911	0.0005
4	1	4	3	0	3	3841.2633	0.0005
4	1	3	3	2	2	4154.4053	0.0003
4	2	3	3	2	2	4275.3450	0.0002
4	1	3	3	1	2	4443.5217	0.0002
4	3	2	3	3	1	4499.0588	-0.0003

3	3	1	2	2	0	4554.3346	-0.0006
4	2	3	3	1	2	4564.4614	0.0001
4	3	1	3	3	0	4682.8048	-0.0006
3	3	0	2	2	1	4697.6185	-0.001
3	2	1	2	1	2	4784.6248	0.0031
4	2	2	3	2	1	4788.2375	-0.0003
5	1	4	4	2	3	5188.7029	-0.0063
5	2	3	4	3	2	5190.6953	0.0007
3	3	0	2	1	1	5195.0277	-0.0002
5	2	4	4	2	3	5226.6627	0.0004
5	1	4	4	1	3	5309.6494	0.0003
5	2	4	4	1	3	5347.6024	0.0001
4	3	2	3	2	1	5474.3439	-0.0001
2	1	1	1	1	0	2382.2971	0.0001
2	0	2	1	0	1	2076.9134	0.0001
3	1	2	2	0	2	4152.2199	0.0001
3	2	1	2	1	1	4180.4536	-0.0001
3	2	2	2	1	2	4372.9273	0.0001
3	3	0	2	2	0	4593.6244	-0.0002
3	3	1	2	2	1	4658.3300	-0.0001
4	3	1	3	2	1	5697.3794	-0.0002
4	1	3	3	0	3	5632.0899	0.0001
4	2	3	3	1	3	5733.5004	0.0002
4	3	2	3	2	2	5886.0385	0.0001
4	4	0	3	3	0	6232.0807	0.0001
4	4	1	3	3	1	6259.1733	0.0001

[a] $\Delta v = v_{exp} - v_{cacl}$.

Table S7. Experimental spectroscopic constants of the nine singly ^{13}C substituted HOMO1[a]

Sub. Species	*A* / MHz	*B* / MHz	*C* / MHz	*N*[b]	*σ* / kHz[b]
C1	826.39601(21)	638.82518(31)	427.32300(7)	12	1.7
C2	831.7216(12)	638.3885(15)	428.72152(4)	10	6.2
C3	829.28112(62)	634.6659(12)	427.21172(23)	11	4.1
C11	834.99631(82)	633.41164(98)	427.99785(31)	13	5.9
C12	836.2286(11)	635.3405(10)	428.26699(45)	11	7.2
C13	832.6839(10)	630.8293(19)	425.58523(39)	11	6.8
C21	827.0727(12)	637.4005(24)	426.81596(56)	9	7.6
C22	833.3091(15)	636.7990(15)	428.38573(45)	11	7.7
C23	833.93125(42)	631.39723(79)	426.60083(18)	11	2.9

[a] All quartic centrifugal distortion constants are fixed at the values of the parent species.
[b] *N* is the number of transitions included and *σ* is the standard deviation of the fit.

Table S8. Experimental transition frequencies of all nine ^{13}C isotopologues of HOMO1.

J'	K_a'	K_c'	J"	K_a"	K_c"	ν_{EXP} / MHz	$\Delta\nu^a$ / MHz
C1							
5	0	5	4	1	4	4560.6962	-0.0030
5	1	5	4	0	4	4568.1030	0.0001
4	0	4	3	1	3	3695.0450	0.0040
4	1	4	3	0	3	3725.5659	-0.0003
3	3	1	2	2	0	4615.2740	0.0009
3	3	0	2	2	1	4757.9405	-0.0009
5	2	4	4	1	3	5251.9210	-0.0001
6	0	6	5	1	5	5417.9248	0.0001
6	1	6	5	0	5	5419.5525	0.0001
4	2	3	3	1	2	4516.1204	-0.0001
3	2	2	2	1	1	3761.1302	0.0001
4	1	3	3	2	2	4028.3505	0.0001
C2							
4	0	4	3	1	3	3705.0300	0.0012
4	1	4	3	0	3	3738.3800	-0.0014
5	0	5	4	1	4	4574.4354	0.0091
3	3	1	2	2	0	4643.9773	0.0008
3	2	2	2	1	1	3781.3005	-0.0022
4	1	3	3	2	2	4023.3769	-0.0001
4	2	3	3	1	2	4539.1907	0.0107
5	2	4	4	1	3	5274.8700	-0.0124
6	0	6	5	1	5	5434.7575	-0.0053
6	1	6	5	0	5	5436.6467	0.0018
C3							
4	0	4	3	1	3	3690.5497	0.0005
4	1	4	3	0	3	3724.9891	-0.0035

5	0	5	4	1	4	4557.2900	0.0083
5	1	5	4	0	4	4565.9746	-0.0092
6	0	6	5	1	5	5414.7263	-0.0007
6	1	6	5	0	5	5416.7230	0.0033
3	3	1	2	2	0	4630.1750	0.0018
3	3	0	2	2	1	4764.4597	-0.0014
5	1	4	4	2	3	5034.5086	-0.0001
5	2	4	4	1	3	5257.2943	0.0001
3	2	2	2	1	1	3769.4517	-0.0001
C11							
4	0	4	3	1	3	3695.4110	0.0156
4	1	4	3	0	3	3733.5090	-0.0095
5	0	5	4	1	4	4564.9081	-0.0001
4	2	3	3	1	2	4546.2748	0.0009
3	3	1	2	2	0	4660.2960	0.0064
3	3	0	2	2	1	4788.8568	-0.0031
5	1	4	4	2	3	5032.1240	-0.0022
6	0	6	5	1	5	5424.3484	0.0021
6	1	6	5	0	5	5426.6981	-0.0010
5	1	5	4	0	4	4574.8577	0.0010
5	2	4	4	1	3	5276.9099	-0.0077
3	2	2	2	1	1	3788.9557	0.0001
4	1	3	3	2	2	3986.5515	0.0001
C12							
5	0	5	4	1	4	4569.2500	0.0117
5	1	5	4	0	4	4578.9100	0.0030
4	0	4	3	1	3	3699.4277	-0.0042
4	1	4	3	0	3	3736.7751	-0.0133
3	3	1	2	2	0	4666.8511	0.0042
3	2	2	2	1	1	3793.4670	0.0069
3	3	0	2	2	1	4797.5771	-0.0029
3	1	3	2	0	2	2920.0757	0.0023
5	2	4	4	1	3	5282.0052	-0.0115
6	0	6	5	1	5	5429.1252	0.0038
5	1	4	4	2	3	5041.6906	0.0001
C13							
6	0	6	5	1	5	5395.3070	0.0035
6	1	6	5	0	5	5397.6770	0.0028
5	0	5	4	1	4	4540.6754	0.0051
4	0	4	3	1	3	3675.9396	0.0125
4	1	4	3	0	3	3714.2130	-0.0092
5	1	5	4	0	4	4550.6721	-0.0072
3	2	2	2	1	1	3774.7760	-0.0047

3	3	1	2	2	0	4646.3260	-0.0046
3	3	0	2	2	1	4774.5947	0.0090
5	2	4	4	1	3	5253.0293	-0.0012
5	1	4	4	2	3	5007.2165	-0.0066
C21							
5	0	5	4	1	4	4555.2755	0.0043
5	1	5	4	0	4	4563.0234	0.0024
4	0	4	3	1	3	3690.2660	-0.0047
6	0	6	5	1	5	5411.5930	-0.0082
3	2	2	2	1	1	3761.6260	-0.0133
3	3	1	2	2	0	4618.3740	0.0028
4	2	3	3	1	2	4515.7480	0.0143
3	3	0	2	2	1	4758.8244	-0.0030
5	1	4	4	2	3	5043.8310	0.0037
C22							
5	0	5	4	1	4	4570.6300	-0.0029
5	1	5	4	0	4	4579.5300	0.0116
4	0	4	3	1	3	3701.3488	-0.0008
4	1	4	3	0	3	3736.3762	-0.0144
6	0	6	5	1	5	5430.4820	-0.0070
6	1	6	5	0	5	5432.5370	0.0059
3	3	1	2	2	0	4651.8930	-0.0059
5	1	4	4	2	3	5049.5120	0.0061
5	2	4	4	1	3	5275.9740	-0.0014
3	2	2	2	1	1	3785.0690	0.0113
4	1	3	3	2	2	4010.5293	-0.0039
C23							
4	0	4	3	1	3	3683.6020	-0.0026
4	1	4	3	0	3	3722.3350	-0.0058
6	1	6	5	0	5	5409.6560	0.0008
3	3	1	2	2	0	4653.6170	-0.0009
3	3	0	2	2	1	4781.0580	-0.0022
5	0	5	4	1	4	4550.5246	0.0028
5	1	5	4	0	4	4560.6842	-0.0020
4	2	3	3	1	2	4536.2630	0.0055
3	2	2	2	1	1	3781.5704	0.0009
5	1	4	4	2	3	5015.3350	0.0018
5	2	4	4	1	3	5263.7410	-0.0001

[a] $\Delta v = v_{exp} - v_{cacl}$.

Table S9. Experimental spectroscopic constants of the nine singly ^{13}C substituted HETERO1[a]

Sub. Species	*A* / MHz	*B* / MHz	*C* / MHz	*N*[b]	*σ* / kHz[b]
C1	802.1575(29)	645.57449(72)	441.68231(28)	11	4.8
C2	806.5321(43)	645.6251(10)	443.02460(48)	9	6.8
C3	803.3460(27)	642.61036(64)	441.21847(26)	10	4.3
C11	807.0302(41)	639.4832(18)	440.32721(34)	10	5.5
C12	810.0304(67)	641.9275(33)	442.32192(67)	7	8.7
C13	804.7948(54)	642.3326(12)	442.74105(53)	10	8.5
C21	810.4912(42)	640.1209(18)	442.08263(46)	8	5.4
C22	810.6288(32)	642.77522(51)	442.76789(23)	8	3.3
C23	805.6379(53)	639.22317(91)	440.22108(36)	8	5.6

[a] All quartic centrifugal distortion constants are fixed at the values of the parent species.
[b] *N* is the number of transitions included and *σ* is the standard deviation of the fit.

Table S10. Experimental transition frequencies of all nine ^{13}C isotopologues of HETERO1.

J'	K_a'	K_c'	J''	K_a''	K_c''	ν_{EXP} / MHz	$\Delta\nu^a$ / MHz
C1							
4	1	4	3	1	3	3798.9760	0.0010
4	0	4	3	0	3	3811.9860	0.0004
5	0	5	4	0	4	4689.8725	0.0019
5	1	5	4	1	4	4686.9010	0.0021
4	2	3	3	2	2	4257.8600	-0.0001
4	1	3	3	1	2	4417.0605	-0.0071
4	2	2	3	2	1	4780.7918	0.0014
5	2	4	4	2	3	5200.0548	-0.0013
5	1	4	4	1	3	5273.8194	0.0064
6	1	6	5	1	5	5571.2106	0.0034
6	0	6	5	0	5	5571.8023	-0.0080
C2							
4	1	4	3	1	3	3809.5830	0.0040
4	2	3	3	2	2	4265.7464	0.0008
4	1	3	3	1	2	4429.2300	-0.0037
5	0	5	4	0	4	4703.7542	0.0110
5	1	5	4	1	4	4700.4761	-0.0026
4	2	2	3	2	1	4783.6706	-0.0008
5	2	4	4	2	3	5212.2542	0.0029
5	1	4	4	1	3	5290.3486	0.0011
6	1	6	5	1	5	5587.5570	-0.0105
C3							
4	0	4	3	0	3	3807.7537	-0.0031
4	2	3	3	2	2	4247.2430	-0.0011
4	1	3	3	1	2	4410.5309	0.0014
5	0	5	4	0	4	4684.3686	-0.0045

5	1	5	4	1	4	4681.0756	-0.0047
4	2	2	3	2	1	4761.8240	0.0008
5	2	4	4	2	3	5190.0683	-0.0015
5	1	4	4	1	3	5268.4184	0.0005
6	0	6	5	0	5	5565.2556	0.0013
6	1	6	5	1	5	5564.5745	0.0085
C11							
4	0	4	3	0	3	3801.6276	-0.0140
4	1	4	3	1	3	3786.1460	0.0007
4	2	3	3	2	2	4234.8055	-0.0009
4	1	3	3	1	2	4404.4990	0.0003
5	0	5	4	0	4	4675.9540	0.0016
5	1	5	4	1	4	4672.1503	0.0018
5	2	4	4	2	3	5178.3173	0.0002
5	1	4	4	1	3	5263.6895	0.0021
6	0	6	5	0	5	5554.8475	0.0023
6	1	6	5	1	5	5554.0196	0.0022
C12							
4	0	4	3	0	3	3818.3050	-0.0090
4	1	4	3	1	3	3802.7491	0.0016
4	2	3	3	2	2	4252.4258	0.0004
4	1	3	3	1	2	4422.6660	-0.0007
5	1	5	4	1	4	4692.7540	-0.0095
5	1	4	4	1	3	5285.8608	0.0023
6	0	6	5	0	5	5579.4724	0.0111
C13							
3	1	2	2	1	1	3460.8106	-0.0005
4	0	4	3	0	3	3818.9607	-0.0007
4	1	4	3	1	3	3804.4798	-0.0186
4	1	3	3	1	2	4418.8968	-0.0046
5	1	5	4	1	4	4695.0010	0.0004
4	2	2	3	2	1	4762.9440	0.0023
5	2	4	4	2	3	5200.5043	-0.0009
5	1	4	4	1	3	5281.0487	0.0052
6	1	6	5	1	5	5581.5959	0.0104
6	0	6	5	0	5	5582.3200	0.0004
C21							
4	1	4	3	1	3	3799.7430	-0.0080
4	0	4	3	0	3	3815.9220	0.0075
4	2	3	3	2	2	4245.9680	0.0006
4	1	3	3	1	2	4418.1870	-0.0030
5	0	5	4	0	4	4693.5090	-0.0023

5	1	5	4	1	4	4689.4750	0.0014
5	2	4	4	2	3	5193.9803	0.0026
5	1	4	4	1	3	5282.3575	0.0005
C22							
4	1	4	3	1	3	3806.6761	-0.0071
4	0	4	3	0	3	3822.1650	0.0005
4	2	3	3	2	2	4257.2540	0.0004
4	1	3	3	1	2	4427.2879	0.0001
5	0	5	4	0	4	4701.3590	0.0012
4	2	2	3	2	1	4765.5340	0.0002
6	0	6	5	0	5	5585.1370	0.0015
6	1	6	5	1	5	5584.3130	0.0014
C23							
4	1	4	3	1	3	3784.8012	-0.0116
4	0	4	3	0	3	3800.0990	0.0005
4	1	3	3	1	2	4401.7270	0.0004
5	0	5	4	0	4	4674.2700	-0.0009
5	1	5	4	1	4	4670.5380	0.0015
4	2	2	3	2	1	4739.0360	0.0006
6	0	6	5	0	5	5552.9827	0.0031
6	1	6	5	1	5	5552.1737	0.0031

[a] $\Delta v = v_{exp} - v_{cacl}$.

Table S11. Kraitchman's coordinates (in Å) and the corresponding *ab initio* values (in Å) at the B3LYP-D3BJ/def2-TZVP level of the nine C atoms in HOMO1 in its principal inertial axis system.

Constants/ Species	*a*		*B*		*c*	
	cal	|kra|	cal	|kra|	cal	|kra|
C1	0.323074	0.1644(94)	2.787510	2.73548(57)	0.364990	0.3728(42)
C2	0.728302	0.5677(20)	1.779970	1.82196(90)	-0.612940	0.6181(26)
C3	2.055187	1.93800(83)	1.812868	2.00988(80)	-1.310283	1.2409(13)
C11	-2.270239	2.37198(68)	0.216516	0.065(25)	1.133463	1.1470(14)
C12	-2.190082	2.11910(77)	-0.518842	0.6393(26)	-0.129270	0.117(13)
C13	-3.292510	3.10606(54)	-1.421769	1.6418(11)	-0.600161	0.6613(26)
C21	1.136971	1.3412(13)	-2.781570	2.66845(67)	-0.275588	0.2628(68)
C22	1.375400	1.5274(11)	-1.617869	1.4952(11)	0.577678	0.5734(29)
C23	2.745546	2.8988(54)	-1.217819	1.0481(15)	1.035986	1.0227(16)

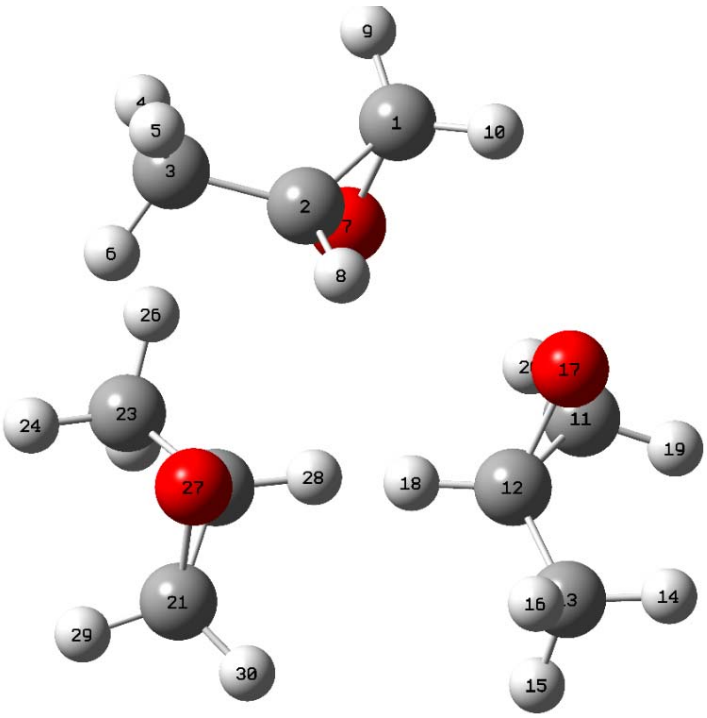

Table S12. Kraitchman's coordinates (in Å) and the corresponding *ab initio* values (in Å) at the B3LYP-D3BJ/def2-TZVP level of the nine C atoms in HETERO1 in its principal inertial axis system.

Constants/	*a*		*B*		*c*	
Species	cal	|kra|	cal	|kra|	cal	|kra|
C1	-0.169189	0.3996(41)	2.771075	2.68146(61)	0.504970	0.5125(32)
C2	0.462181	0.3302(54)	1.928137	1.94124(92)	-0.508248	0.5042(35)
C3	1.809693	1.71364(94)	2.234212	2.36706(69)	-1.089946	1.0093(16)
C11	2.567216	2.72305(66)	-2.059416	1.86069(99)	-0.552845	0.5754(32)
C12	2.065940	2.1455(11)	-1.174212	1.0442(22)	0.498459	0.4997(46)
C13	1.232522	1.3306(14)	-1.670314	1.6771(11)	1.641632	1.6136(12)
C21	-2.436594	2.49481(73)	-0.126588	0.2861(65)	0.947152	0.9399(20)
C22	-2.000285	1.93679(85)	-0.827134	0.8896(19)	-0.259738	0.2701(61)
C23	-2.820445	2.67317(69)	-1.910294	1.99854(95)	-0.896489	0.9542(20)

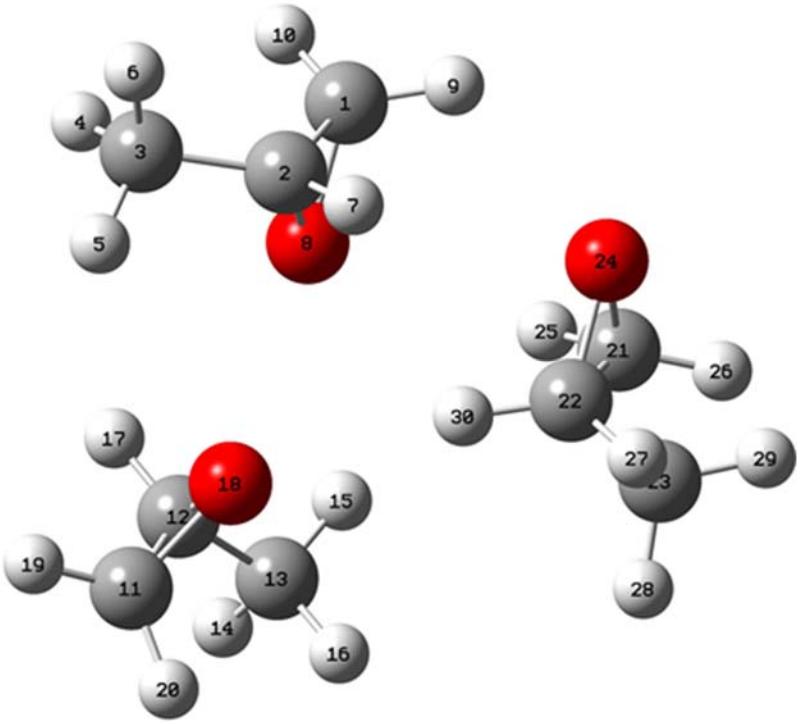

Point S2. The Python program for extracting the heavy atom frame using the ^{13}C isotopic data.

A python program was written to aid the extraction of the heavy atom frame using the absolute Cartesian coordinates obtained using the ^{13}C isotopic data. It is available upon requested from the authors. It consists of three main steps.

Step 1. Group the C atoms to form three PO subunits
Each x, y and z coordinates from the Kraitchman's substitution procedure can take on "+" or "-" signs. From these combinations, possible C-C distances are calculated. Suitable C atoms to make up a PO subunit should have one ring C-C distance and one side chain C-C distance in the range of 1.45-1.47 and 1.49-1.54, respectively., In addition, the third C-C distance should be in the range of 2.55-2.63 Å.

These criteria were first tested on HOMO1 and then used for the HETERO1 search. It is important to test these thresholds. As mentioned in the main text, each C atom has three Cartesian coordinates, i.e. x, y, and z, and for each x, y or z, we have two possible signs, leading to 2 x 2 x 2 = 8 possible positions. Since the starting C atom of each PO unit can take on any one of the eight possible Cartesian coordinates, one would expect eight possible sets of Cartesian coordinates for each PO unit, keeping in mind that the two further C atoms have to choose their Cartesian coordinates based on the first C atom. In total, we have 3 PO subunits and would expect 24 sets of the "three-carbon-atom" groups. If the threshold is set too tight, this would result in fewer possible sets of the "three-carbon-atom" groups. For example, one may obtain only 8 sets which correspond to only one PO subunit. If the threshold is set too loose, one may obtain some "extra" sets which contain fake (or improper) PO subunit(s). We found that a good approach is to use the related HOMO1 distances and then slowly expanded the thresholds until we got the expected 24 sets of the "three-carbon-atom" groups.

Step 2. Add O and H atoms to each PO subunit and assemble a reasonable PO trimer structure

First, we put the O and H atoms back into the carbon frames identified in Step 1 to complete the PO subunits. As mentioned in Step 1, the 24 sets of Cartesian coordinates obtained for the "three-carbon-atom" groups actually correspond to 3 PO subunits where each subunit has 8 possible sets of Cartesian coordinates. For the PO trimer, we have 8x8x8 (i.e. 512) Cartesian coordinate combinations in total.

Second, since overlapping/overcrowding of the PO subunits is not physically sensible in the trimer, we utilized this requirement to further reduce the number of possible trimer combinations. To do this, one needs to set a minimum acceptable separation for all *intermolecular* distances. Generally, we found that this criterion is not as strict as those thresholds set in Step 1. As along as this threshold is set to any value between 2 to 3 Å, the possible combination is reduced from 512 to 8. On the other hand, if the threshold is set to a value between 0 to 2 Å, all 512 combinations remain. This basically means that we did not control the overlapping/overcrowding of the PO subunits. If the threshold is set to a value larger than 3 Å, no combination was obtained. This is because the substitution coordinates place a constraint on the 512 Cartesian combinations and do not allow PO subunits to be so far away from each other.

The eight sets of possible Cartesian coordinates identified for the PO trimer correspond actually to just one PO trimer structure if we count the mirror-image structures as the same.

Step 3. DFT geometry optimization
The HETERO1 geometry obtained above was used as the starting point for a DFT geometry optimization to obtain the HETERO1 structure that corresponds to the experimental spectrum observed. See the main text for further discussion on the vibrational corrections to the equilibrium structure.

Point S3. Computational details on vibrational corrections

In many rotational spectroscopic research papers, the experimental rotational constants in the ground vibrational state, also called effective rotational constants, are directly compared to the equilibrium rotational constants obtained from ab initio calculations. For fully quantitative purposes, the difference between the two quantities, issuing from the vibration-rotation interaction constants ruled by semi-diagonal cubic force constants [S1,S2], however, cannot be neglected. The availability of effective codes for the computation of analytical harmonic frequencies for double-hybrid functionals [S3] and of effective numerical evaluations of third- and fourth-energy derivatives [S2] allows, in principle, the computation of accurate ground state rotational constants including vibrational corrections, providing full reconciliation between accuracy and interpretability in the challenging field of large non-covalent complexes.

All calculations on vibrational corrections were performed with Gaussian16 suite of programs [S4]. The six lower energy structures from the conformational search were further refined by employing the B2PLYP [S5] (B2) and the DSD-PBEP86 [S6] (rPP) double hybrid functionals in conjunction with the jun-cc-pVTZ [S7,S8]. In all calculation the empirical dispersion (D3BJ) [S9] were included. Geometry optimizations were performed with tight convergence criteria (i.e., 1×10^{-5} hartree/bohr and 4×10^{-5} bohr on RMS force and displacements, respectively, with maximum values being 1.5 times larger).

Harmonic and anharmonic calculations were carried out at B3LYP-D3BJ [S9,S10] / jun-cc-pVDZ [S7,S8] level of theory. Harmonic force fields were obtained using analytic derivatives of energy and transition moments, whereas higher-order derivatives were computed through numerical differentiation using a step of 0.01 amu$^{1/2}$ Å for the displacements along the mass-weighted normal coordinates. Equilibrium rotational constants (B_x^e, *x=a,b,c*) were derived from the B2 and rPP optimized geometries, while, in order to obtain rotational constants of the vibrational ground-state (B_x^0), the vibrational corrections were computed from the vibration-rotation interaction constants (α_k^x) obtained within the vibrational perturbation theory (VPT2) at B3 level according to the following equation:

$$B_x^0 = B_x^e - \frac{1}{2}\sum_{k=1}^{3N-6} \alpha_k^x$$

Within the same composite scheme, the equilibrium and vibrational ground-state dipole moments were also computed.

The electronic contribution to the corrections has been neglected, since it contributes less than 0.05% with respect to the vibrational corrections [S10].

Bibliography

[S1] I. M. Mills in Molecular Spectroscopy: Modern Research, ed. K.N. Rao and C.W. Mathews, Academic Press, **1972**.

[S2] V. Barone, *J. Chem. Phys.* **2005**, *122*, 014108/1-10.

[S3] M. Biczysko, G. Scalmani, J. Bloino, V. Barone, *J. Chem. Theory Comput.* **2010**, *6*, 2115-2125.

[S4] Gaussian 16, Revision C.01, M. J. Frisch, *et al.* Gaussian, Inc., Wallingford CT, **2016**. See page S27 for the full reference

[S5] a) S. Grimme, J. Chem. Phys. **2006**, *124*, 034108/1-16; b) A. D. Becke, *J. Chem. Phys.* **1993**, *98*, 5648-5652.

[S6] G. Santra, N. Sylvetsky, J. M. L. Martin, *J. Chem. Theory Comput.* **2019**, *123*, 5129-5143.

[S7] T. H. Dunning Jr., *J. Chem. Phys.* **1989**, *90*, 1007.

[S8] E. Papajak, H. R. Leverentz, J. Zheng, D. G. Truhlar, *J. Chem. Theory Comput.* **2009**, *5*, 1197-1202.

[S9] S. Grimme, S. Ehrlich, L. Goerigk, *J. Comput. Chem.* **2011**, *32*, 1456-1465.

[S10] M. Piccardo, E. Penocchio, C. Puzzarini, M. Biczysko, V. Barone, *J. Phys. Chem. A* **2015**, *119*, 10, 2058-2082.

Table S13. Rotational constants (MHz) of HOMO and HETERO complexes. Equilibrium geometries, at B2PLYPD3/jun-cc-pVTZ (B2) level of theory; vibrational correction at B3LYP/jun-cc-pVDZ + empirical dispersion D3BJ (B3) level of theory.

	Ae	*Be*	*Ce*	*vibA0*	*vibB0*	*vibC0*	*A0*	*B0*	*C0*
HOMO1	*842.195*	*649.778*	*434.669*	*-13.333*	*-10.377*	*-7.601*	828.862	639.401	427.068
HOMO2	*795.369*	*642.248*	*471.165*	*-12.060*	*-11.182*	*-11.499*	783.309	631.066	459.666
HOMO3	*790.169*	*656.189*	*424.418*	*-8.819*	*-11.417*	*-7.139*	781.350	644.772	417.279
HOMO4	*767.152*	*655.671*	*444.383*	*-13.441*	*-10.033*	*-9.133*	753.711	645.638	435.250
HOMO5	*801.240*	*618.221*	*448.915*	*-13.809*	*-11.672*	*-10.774*	787.431	606.549	438.141
HOMO6	*762.179*	*672.498*	*396.359*	*-11.225*	*-9.468*	*-5.600*	750.954	663.030	390.759
HETERO1	*816.209*	*656.550*	*452.306*	*-14.744*	*-10.511*	*-9.373*	801.465	646.039	442.933
HETERO1o	*733.845*	*685.917*	*441.625*	*-11.483*	*-11.057*	*-8.259*	722.362	674.860	433.366
HETERO2	*748.796*	*677.420*	*455.350*	*-10.766*	*-13.236*	*-10.321*	738.030	664.184	445.029
HETERO3	*813.402*	*602.213*	*463.960*	*-10.630*	*-8.221*	*-8.191*	802.772	593.992	455.769
HETERO4	*887.267*	*579.747*	*441.027*	*-16.523*	*-9.744*	*-9.471*	870.744	570.003	431.556
HETERO5	*791.026*	*675.544*	*437.733*	*-7.426*	*-11.389*	*-6.381*	783.600	664.155	431.352

Table S14. Rotational constants (MHz) of HOMO and HETERO complexes. Equilibrium geometries, at DSD-PBEP86-D3BJ/jun-cc-pVTZ (rPP) level of theory; vibrational correction at B3LYP/jun-cc-pVDZ + empirical dispersion D3BJ (B3) level of theory.

	Ae	*Be*	*Ce*	*vibA0*	*vibB0*	*vibC0*	*A0*	*B0*	*C0*
HOMO1	*848.642*	*648.445*	*438.418*	*-13.333*	*-10.377*	*-7.601*	835.309	638.068	430.817
HOMO2	*795.778*	*639.697*	*468.845*	*-12.060*	*-11.182*	*-11.499*	783.718	628.515	457.346
HOMO3	*796.206*	*656.791*	*424.845*	*-8.819*	*-11.417*	*-7.139*	787.387	645.374	417.706
HOMO4	*763.303*	*653.939*	*441.867*	*-13.441*	*-10.033*	*-9.133*	749.862	643.906	432.734
HOMO5	*802.173*	*616.057*	*447.774*	*-13.809*	*-11.672*	*-10.774*	788.364	604.385	437.000
HOMO6	*761.905*	*673.655*	*397.195*	*-11.225*	*-9.468*	*-5.600*	750.680	664.187	391.595
HETERO1	*824.591*	*655.451*	*452.751*	*-14.744*	*-10.511*	*-9.373*	809.847	644.940	443.378
HETERO1o	*742.342*	*679.443*	*443.092*	*-11.483*	*-11.057*	*-8.259*	730.859	668.386	434.833
HETERO2	*747.707*	*675.017*	*452.402*	*-10.766*	*-13.236*	*-10.321*	736.941	661.781	442.081
HETERO3	*823.651*	*599.809*	*462.276*	*-10.630*	*-8.221*	*-8.191*	813.021	591.588	454.085
HETERO4	*886.032*	*576.825*	*438.744*	*-16.523*	*-9.744*	*-9.471*	869.509	567.081	429.273
HETERO5	*801.509*	*670.080*	*443.173*	*-7.426*	*-11.389*	*-6.381*	794.083	658.691	436.792

Table 15. Results: dipole moments of HOMO and HETERO complexes (Debye) in Principal Axis frame. dipole moments at B2PLYPD3/jun-cc-pVTZ (B2) level of theory; vibrational correction at B3LYP/jun-cc-pVDZ + empirical dispersion D3BJ (B3) level of theory.

	$\mu_a e$	$\mu_b e$	$\mu_c e$	$\Delta\mu_a 0$	$\Delta\mu_b 0$	$\Delta\mu_c 0$
HOMO1	*0.7527*	*-1.5136*	*0.8259*	*-0.0164*	*0.0361*	*-0.0270*
HOMO2	*-1.1450*	*-0.2357*	*-1.4072*	*0.0081*	*0.0230*	*-0.0076*
HOMO3	*0.9717*	*-1.1374*	*-1.0459*	*-0.0260*	*0.0260*	*0.0107*
HOMO4	*-0.9640*	*0.1983*	*1.4650*	*-0.0188*	*-0.0675*	*-0.0557*
HOMO5	*0.1994*	*-1.2975*	*-0.1122*	*0.0568*	*-0.0565*	*-0.0374*
HOMO6	*-1.4628*	*0.0076*	*1.0764*	*0.0178*	*-0.0072*	*-0.0591*
HETERO1	*1.6342*	*-0.9718*	*0.7500*	*-0.0534*	*0.0451*	*0.0093*
HETERO1o	*-0.1267*	*1.8539*	*0.7944*	*0.0075*	*-0.0227*	*-0.0265*
HETERO2	*1.2274*	*-0.0333*	*1.1976*	*-0.0582*	*0.0239*	*0.0311*
HETERO3	*1.4732*	*-0.4987*	*0.9978*	*-0.0273*	*0.0100*	*-0.0098*
HETERO4	*-1.1362*	*-0.4189*	*-1.1145*	*-0.0318*	*0.0409*	*0.0372*
HETERO5	*-1.1400*	*-0.5574*	*-1.1858*	*-0.0142*	*-0.0877*	*0.0300*

Table S16. Results: dipole moments of HOMO and HETERO complexes (Debye) in Principal Axis frame. dipole moments at DSD-PBEP86-D3BJ/jun-cc-pVTZ (rPP) level of theory; vibrational correction at B3LYP/jun-cc-pVDZ + empirical dispersion D3BJ (B3) level of theory.

	$\mu_a e$	$\mu_b e$	$\mu_c e$	$\Delta\mu_a 0$	$\Delta\mu_b 0$	$\Delta\mu_c 0$
HOMO1	*0.7837*	*-1.4584*	*0.8518*	*-0.0164*	*0.0361*	*-0.0270*
HOMO2	*1.1341*	*-0.2502*	*1.4118*	*0.0081*	*0.0230*	*-0.0076*
HOMO3	*-1.0319*	*-1.1153*	*1.0783*	*-0.0260*	*0.0260*	*0.0107*
HOMO4	*0.8990*	*0.2360*	*-1.4407*	*-0.0188*	*-0.0675*	*-0.0557*
HOMO5	*-0.2130*	*-1.3258*	*0.1105*	*0.0568*	*-0.0565*	*-0.0374*
HOMO6	*-1.4366*	*0.0317*	*1.0835*	*0.0178*	*-0.0072*	*-0.0591*
HETERO1	*1.6256*	*-0.9096*	*0.7876*	*-0.0534*	*0.0451*	*0.0093*
HETERO1o	*0.0181*	*1.9177*	*0.7665*	*0.0075*	*-0.0227*	*-0.0265*
HETERO2	*1.1720*	*-0.0995*	*1.2393*	*-0.0582*	*0.0239*	*0.0311*
HETERO3	*1.4435*	*-0.5495*	*0.9540*	*-0.0273*	*0.0100*	*-0.0098*
HETERO4	*-1.1263*	*-0.4513*	*-1.0959*	*-0.0318*	*0.0409*	*0.0372*
HETERO5	*-1.2230*	*-0.5149*	*-1.1837*	*-0.0142*	*-0.0877*	*0.0300*

Table S17. Absolute Energies of HOMO and HETERO most stable conformers and relative stabilities of the HOMO and HETERO complexes (kJ mol^{-1}). Within parenthesis, the relative stabilities between the HOMO and HETERO most stable conformers are given. Energies, at B2PLYPD3/jun-cc-pVTZ (B2), harmonic zero point vibrational energy at B3LYP/jun-cc-pVDZ + empirical dispersion D3BJ (B3) level of theory.

	E	*ΔE*	*E* +*ZPVE(Harm)*	*ΔE* +*ZPVE(Harm)*
HOMO1	*-1520408.700*	*0.000 (0.000)*	*-1519734.828*	*0.000 (0.000)*
HOMO2		*0.196*		*1.512*
HOMO3		*0.872*		*1.696*
HOMO4		*1.528*		*1.862*
HOMO5		*0.995*		*1.077*
HOMO6		*1.583*		*1.585*
HETERO1	*-1520408.590*	*0.000 (0.111)*	*-1519734.791*	*0.000 (0.037)*
HETERO1o		*0.017*		*0.650*
HETERO2		*0.191*		*0.503*
HETERO3		*1.002*		*1.145*
HETERO4		*0.689*		*1.606*
HETERO5		*1.014*		*1.412*

Table S18. Absolute Energies of HOMO and HETERO most stable conformers and relative stabilities of the HOMO and HETERO complexes (kJ mol^{-1}). Within parenthesis, the relative stabilities between the HOMO and HETERO most stable conformers are given. Energies, at DSD-PBEP86-D3BJ/jun-cc-pVTZ (rPP), harmonic zero point vibrational energy at B3LYP-D3BJ/jun-cc-pVDZ (B3) level of theory.

	E	*ΔE*	*E* +*ZPVE(Harm)*	*ΔE* +*ZPVE(Harm)*
HOMO1	*-1518879.746*	*0.000 (0.006)*	*-1518205.874*	*0.000 (0.079)*
HOMO2		*0.779*		*2.095*
HOMO3		*1.071*		*1.895*
HOMO4		*2.306*		*2.640*
HOMO5		*1.362*		*1.444*
HOMO6		*1.821*		*1.823*
HETERO1	*-1518879.752*	*0.000 (0.000)*	*-1518205.953*	*0.000 (0.000)*
HETERO1o		*0.931*		*1.564*
HETERO2		*1.047*		*1.360*
HETERO3		*1.849*		*1.992*
HETERO4		*1.542*		*2.460*
HETERO5		*1.588*		*1.987*

Table S19. HOMO1 ^{13}C isotolologues: equi. B2, vib. B3 level of theory (MHz)

	Ae	*Be*	*Ce*	*vibA0*	*vibB0*	*vibC0*	*A0*	*B0*	*C0*
HOMO1	*842.195*	*649.778*	*434.669*	*-13.333*	*-10.377*	*-7.601*	*828.862*	*639.401*	*427.068*
C1	*840.353*	*644.221*	*435.495*	*-12.646*	*-10.260*	*-7.434*	*827.707*	*633.961*	*428.061*
C2	*837.174*	*649.120*	*436.169*	*-13.483*	*-10.181*	*-7.517*	*823.691*	*638.939*	*428.652*
C3	*834.927*	*645.154*	*434.669*	*-14.116*	*-9.876*	*-7.680*	*820.811*	*635.278*	*426.989*
C11	*840.353*	*644.221*	*435.495*	*-14.373*	*-10.377*	*-7.737*	*825.980*	*633.844*	*427.758*
C12	*841.685*	*645.969*	*435.680*	*-13.394*	*-10.259*	*-7.537*	*828.291*	*635.710*	*428.143*
C13	*838.433*	*641.085*	*432.885*	*-14.505*	*-9.680*	*-7.303*	*823.928*	*631.405*	*425.582*
C21	*832.030*	*648.350*	*434.188*	*-13.421*	*-10.299*	*-7.635*	*818.609*	*638.051*	*426.553*
C22	*838.490*	*647.689*	*435.840*	*-13.150*	*-9.892*	*-7.338*	*825.340*	*637.797*	*428.502*
C23	*839.137*	*642.276*	*434.075*	*-13.981*	*-10.027*	*-7.537*	*825.156*	*632.249*	*426.538*

Table S20. Electric Dipole Moment HOMO1 ^{13}C isotolologues: equi. B2, vib. B3 level of theory (Debye) in Principal Axis frame

	$\mu_a e$	$\mu_b e$	$\mu_c e$	$\Delta\mu_a 0$	$\Delta\mu_b 0$	$\Delta\mu_c 0$
HOMO1	*0.7527*	*-1.5136*	*0.8259*	*-0.0164*	*0.0361*	*-0.0270*
C1	*0.7492*	*-1.5132*	*0.8298*	*-0.0187*	*0.0361*	*-0.0280*
C2	*0.7422*	*-1.5209*	*0.8219*	*-0.0171*	*0.0250*	*-0.0333*
C3	*0.7163*	*-1.5346*	*0.8195*	*-0.0129*	*0.0283*	*-0.0282*
C11	*0.7508*	*-1.5123*	*0.8299*	*-0.0189*	*0.0324*	*-0.0358*
C12	*0.7424*	*-1.5188*	*0.8258*	*-0.0135*	*0.0334*	*-0.0323*
C13	*0.7154*	*-1.5310*	*0.8271*	*-0.0178*	*0.0312*	*-0.0350*
C21	*0.7819*	*-1.4970*	*0.8291*	*-0.0202*	*0.0551*	*-0.0137*
C22	*0.7730*	*-1.5059*	*0.8212*	*-0.0140*	*0.0459*	*-0.0192*
C23	*0.7823*	*-1.5031*	*0.8175*	*-0.0228*	*0.0380*	*-0.0212*

Table S21. HOMO1 ^{13}C isotolologues: equi. rPP, vib. B3 level of theory (MHz)

	Ae	*Be*	*Ce*	*vibA0*	*vibB0*	*vibC0*	*A0*	*B0*	*C0*
HOMO1	*848.642*	*648.445*	*438.418*	*-13.333*	*-10.377*	*-7.601*	*835.309*	*638.068*	*430.817*
C1	*837.952*	*648.334*	*435.596*	*-12.646*	*-10.260*	*-7.434*	*825.306*	*638.074*	*428.162*
C2	*843.492*	*647.832*	*437.057*	*-13.483*	*-10.181*	*-7.517*	*830.009*	*637.651*	*429.540*
C3	*841.057*	*644.008*	*435.514*	*-14.116*	*-9.876*	*-7.680*	*826.941*	*634.132*	*427.834*
C11	*846.774*	*642.755*	*436.300*	*-14.373*	*-10.377*	*-7.737*	*832.401*	*632.378*	*428.563*
C12	*848.057*	*644.740*	*436.576*	*-13.394*	*-10.259*	*-7.537*	*834.663*	*634.481*	*429.039*
C13	*844.440*	*640.140*	*433.826*	*-14.505*	*-9.680*	*-7.303*	*829.935*	*630.460*	*426.523*
C21	*838.618*	*646.843*	*435.057*	*-13.421*	*-10.299*	*-7.635*	*825.197*	*636.544*	*427.422*
C22	*845.052*	*646.253*	*436.711*	*-13.150*	*-9.892*	*-7.338*	*831.902*	*636.361*	*429.373*
C23	*845.701*	*640.799*	*434.925*	*-13.981*	*-10.027*	*-7.537*	*831.720*	*630.772*	*427.388*

Table S22. Electric Dipole Moment HOMO1 ^{13}C isotolologues: equi. rPP, vib. B3 level of theory (Debye) in Principal Axis frame

	$\mu_a e$	$\mu_b e$	$\mu_c e$	$\Delta\mu_a 0$	$\Delta\mu_b 0$	$\Delta\mu_c 0$
HOMO1	*0.7837*	*-1.4584*	*0.8518*	*-0.0164*	*0.0361*	*-0.0270*
C1	*0.7826*	*1.4567*	*-0.8555*	*-0.0187*	*0.0361*	*-0.0280*
C2	*0.7747*	*1.4654*	*-0.8479*	*-0.0171*	*0.0250*	*-0.0333*
C3	*0.7493*	*1.4799*	*-0.8456*	*-0.0129*	*0.0283*	*-0.0282*
C11	*0.7807*	*1.4576*	*-0.8558*	*-0.0189*	*0.0324*	*-0.0358*
C12	*0.7736*	*1.4638*	*-0.8517*	*-0.0135*	*0.0334*	*-0.0323*
C13	*0.7481*	*1.4762*	*-0.8531*	*-0.0178*	*0.0312*	*-0.0350*
C21	*0.8121*	*1.4408*	*-0.8550*	*-0.0202*	*0.0551*	*-0.0137*
C22	*0.8029*	*1.4506*	*-0.8472*	*-0.0140*	*0.0459*	*-0.0192*
C23	*0.8106*	*1.4484*	*-0.8435*	*-0.0228*	*0.0380*	*-0.0212*

Table S23. HOMO2 ^{13}C isotolologues: equi. B2, vib. B3 level of theory (MHz)

	Ae	*Be*	*Ce*	*vibA0*	*vibB0*	*vibC0*	*A0*	*B0*	*C0*
HOMO2	*795.369*	*642.248*	*471.165*	*-12.060*	*-11.182*	*-11.499*	*783.309*	*631.066*	*459.666*
C1	*785.530*	*644.831*	*471.717*	*-11.175*	*-13.300*	*-10.952*	*774.355*	*631.531*	*460.765*
C2	*790.919*	*645.825*	*473.242*	*-11.437*	*-13.296*	*-11.115*	*779.482*	*632.529*	*462.127*
C3	*786.877*	*644.250*	*472.314*	*-11.291*	*-13.210*	*-10.848*	*775.586*	*631.040*	*461.466*
C11	*792.258*	*642.886*	*473.220*	*-12.262*	*-13.469*	*-11.426*	*779.996*	*629.417*	*461.794*
C12	*793.734*	*640.523*	*471.359*	*-12.428*	*-12.711*	*-11.240*	*781.306*	*627.812*	*460.119*
C13	*792.506*	*640.278*	*472.408*	*-11.815*	*-13.000*	*-10.989*	*780.691*	*627.278*	*461.419*
C21	*795.802*	*639.144*	*471.367*	*-11.737*	*-12.780*	*-10.899*	*784.065*	*626.364*	*460.468*
C22	*792.747*	*641.107*	*471.333*	*-11.151*	*-12.975*	*-10.577*	*781.596*	*628.132*	*460.756*
C23	*789.528*	*642.840*	*472.457*	*-12.085*	*-13.059*	*-11.130*	*777.443*	*629.781*	*461.327*

Table S24. Electric Dipole Moment HOMO2 ^{13}C isotolologues: equi. B2, vib. B3 level of theory (Debye) in Principal Axis frame

	$\mu_a e$	$\mu_b e$	$\mu_c e$	$\Delta\mu_a 0$	$\Delta\mu_b 0$	$\Delta\mu_c 0$
HOMO2	*-1.1450*	*-0.2357*	*-1.4072*	*0.0081*	*0.0230*	*-0.0076*
C1	*-1.1519*	*-0.1906*	*-1.4084*	*0.0159*	*0.0106*	*-0.0070*
C2	*-1.1528*	*-0.2101*	*-1.4048*	*0.0108*	*0.0180*	*-0.0107*
C3	*-1.1465*	*-0.2408*	*-1.4051*	*0.0014*	*0.0198*	*-0.0059*
C11	*-1.1545*	*-0.2217*	*-1.4016*	*0.0137*	*0.0153*	*-0.0088*
C12	*-1.1471*	*-0.2389*	*-1.4049*	*0.0088*	*0.0202*	*-0.0144*
C13	*-1.1386*	*-0.2360*	*-1.4123*	*0.0111*	*0.0229*	*-0.0122*
C21	*-1.1511*	*-0.2072*	*-1.4067*	*0.0094*	*0.0215*	*-0.0070*
C22	*-1.1577*	*-0.1840*	*-1.4045*	*0.0065*	*0.0261*	*-0.0082*
C23	*-1.1618*	*-0.2024*	*-1.3985*	*0.0103*	*0.0275*	*-0.0121*

Table S25. HOMO2 ^{13}C isotolologues: equi. rPP, vib. B3 level of theory (MHz)

	Ae	*Be*	*Ce*	*vibA0*	*vibB0*	*vibC0*	*A0*	*B0*	*C0*
HOMO2	*795.778*	*639.697*	*468.845*	*-12.060*	*-11.182*	*-11.499*	*783.718*	*628.515*	*457.346*
C1	*785.136*	*638.705*	*465.585*	*-11.175*	*-13.300*	*-10.952*	*773.961*	*625.405*	*454.633*
C2	*790.720*	*639.639*	*467.083*	*-11.437*	*-13.296*	*-11.115*	*779.283*	*626.343*	*455.968*
C3	*786.799*	*637.936*	*466.088*	*-11.291*	*-13.210*	*-10.848*	*775.508*	*624.726*	*455.240*
C11	*792.300*	*636.605*	*467.038*	*-12.262*	*-13.469*	*-11.426*	*780.038*	*623.136*	*455.612*
C12	*793.795*	*634.142*	*465.177*	*-12.428*	*-12.711*	*-11.240*	*781.367*	*621.431*	*453.937*
C13	*792.578*	*633.931*	*466.191*	*-11.815*	*-13.000*	*-10.989*	*780.763*	*620.931*	*455.202*
C21	*795.568*	*632.994*	*465.188*	*-11.737*	*-12.780*	*-10.899*	*783.831*	*620.214*	*454.289*
C22	*792.239*	*635.189*	*465.209*	*-11.151*	*-12.975*	*-10.577*	*781.088*	*622.214*	*454.632*
C23	*789.203*	*636.865*	*466.340*	*-12.085*	*-13.059*	*-11.130*	*777.118*	*623.806*	*455.210*

Table 26. Electric Dipole Moment HOMO2 ^{13}C isotolologues: equi. rPP, vib. B3 level of theory (Debye) in Principal Axis frame

	$\mu_a e$	$\mu_b e$	$\mu_c e$	$\Delta\mu_a 0$	$\Delta\mu_b 0$	$\Delta\mu_c 0$
HOMO2	*1.1341*	*-0.2502*	*1.4118*	*0.0081*	*0.0230*	*-0.0076*
C1	*1.1344*	*-0.2301*	*1.4150*	*0.0159*	*0.0106*	*-0.0070*
C2	*1.1349*	*-0.2491*	*1.4114*	*0.0108*	*0.0180*	*-0.0107*
C3	*1.1276*	*-0.2789*	*1.4116*	*0.0014*	*0.0198*	*-0.0059*
C11	*1.1365*	*-0.2581*	*1.4084*	*0.0137*	*0.0153*	*-0.0088*
C12	*1.1291*	*-0.2736*	*1.4115*	*0.0088*	*0.0202*	*-0.0144*
C13	*1.1209*	*-0.2704*	*1.4186*	*0.0111*	*0.0229*	*-0.0122*
C21	*1.1342*	*-0.2422*	*1.4131*	*0.0094*	*0.0215*	*-0.0070*
C22	*1.1409*	*-0.2220*	*1.4110*	*0.0065*	*0.0261*	*-0.0082*
C23	*1.1441*	*-0.2414*	*1.4053*	*0.0103*	*0.0275*	*-0.0121*

Table 27. HETERO1 ^{13}C isotolologues: equi. B2, vib. B3 level of theory (MHz)

	Ae	*Be*	*Ce*	*vibA0*	*vibB0*	*vibC0*	*A0*	*B0*	*C0*
HETERO1	*816.209*	*656.550*	*452.306*	*-14.744*	*-10.511*	*-9.373*	*801.465*	*646.039*	*442.933*
C1	*806.369*	*656.237*	*449.318*	*-14.445*	*-10.610*	*-9.277*	*791.924*	*645.627*	*440.041*
C2	*811.001*	*656.211*	*450.746*	*-14.469*	*-10.623*	*-9.295*	*796.532*	*645.588*	*441.451*
C3	*807.885*	*653.072*	*448.940*	*-14.135*	*-10.637*	*-9.271*	*793.750*	*642.435*	*439.669*
C11	*811.280*	*650.088*	*447.964*	*-13.511*	*-11.190*	*-9.066*	*797.769*	*638.898*	*438.898*
C12	*814.430*	*652.513*	*450.039*	*-14.678*	*-11.420*	*-9.636*	*799.752*	*641.093*	*440.403*
C13	*809.312*	*652.825*	*450.522*	*-14.590*	*-10.967*	*-9.490*	*794.722*	*641.858*	*441.032*
C21	*814.952*	*650.656*	*449.830*	*-14.989*	*-10.363*	*-9.188*	*799.963*	*640.293*	*440.642*
C22	*815.119*	*653.285*	*450.468*	*-13.662*	*-11.220*	*-9.419*	*801.457*	*642.065*	*441.049*
C23	*810.095*	*649.630*	*447.842*	*-14.188*	*-10.530*	*-9.192*	*795.907*	*639.100*	*438.650*

Table S28. Electric Dipole Moment HETERO1 ^{13}C isotolologues: equi. B2, vib. B3 level of theory (Debye) in Principal Axis frame

	$\mu_a e$	$\mu_b e$	$\mu_c e$	$\Delta\mu_a 0$	$\Delta\mu_b 0$	$\Delta\mu_c 0$
HETERO1	*1.6342*	*-0.9718*	*0.7500*	*-0.0534*	*0.0451*	*0.0093*
C1	*1.6400*	*-0.9586*	*0.7543*	*-0.0567*	*0.0597*	*0.0136*
C2	*1.6292*	*-0.9818*	*0.7478*	*-0.0513*	*0.0535*	*0.0096*
C3	*1.6047*	*-1.0205*	*0.7490*	*-0.0425*	*0.0603*	*0.0075*
C11	*1.6632*	*-0.9150*	*0.7576*	*-0.0507*	*0.0400*	*0.0039*
C12	*1.6499*	*-0.9489*	*0.7450*	*-0.0464*	*0.0437*	*0.0033*
C13	*1.6513*	*-0.9538*	*0.7355*	*-0.0398*	*0.0435*	*-0.0018*
C21	*1.6269*	*-0.9787*	*0.7569*	*-0.0473*	*0.0492*	*0.0026*
C22	*1.6240*	*-0.9895*	*0.7490*	*-0.0510*	*0.0560*	*0.0094*
C23	*1.6034*	*-1.0240*	*0.7470*	*-0.0540*	*0.0486*	*0.0050*

Table 29. HETERO1 ^{13}C isotolologues: equi. rPP, vib. B3 level of theory (MHz)

	Ae	*Be*	*Ce*	*vibA0*	*vibB0*	*vibC0*	*A0*	*B0*	*C0*
HETERO1	*824.629*	*655.418*	*452.741*	*-14.744*	*-10.511*	*-9.373*	*809.885*	*644.907*	*443.368*
C1	*814.780*	*655.073*	*449.798*	*-14.445*	*-10.610*	*-9.277*	*800.335*	*644.463*	*440.521*
C2	*819.332*	*655.092*	*451.191*	*-14.469*	*-10.623*	*-9.295*	*804.863*	*644.469*	*441.896*
C3	*816.065*	*652.049*	*449.335*	*-14.135*	*-10.637*	*-9.271*	*801.930*	*641.412*	*440.064*
C11	*819.896*	*648.772*	*448.380*	*-13.511*	*-11.190*	*-9.066*	*806.385*	*637.582*	*439.314*
C12	*822.873*	*651.371*	*450.489*	*-14.678*	*-11.420*	*-9.636*	*808.195*	*639.951*	*440.853*
C13	*817.577*	*651.765*	*450.936*	*-14.590*	*-10.967*	*-9.490*	*802.987*	*640.798*	*441.446*
C21	*823.357*	*649.441*	*450.204*	*-14.989*	*-10.363*	*-9.188*	*808.368*	*639.078*	*441.016*
C22	*823.502*	*652.213*	*450.923*	*-13.662*	*-11.220*	*-9.419*	*809.840*	*640.993*	*441.504*
C23	*818.220*	*648.729*	*448.346*	*-14.188*	*-10.530*	*-9.192*	*804.032*	*638.199*	*439.154*

Table S30. Electric Dipole Moment HETERO1 ^{13}C isotolologues: equi. rPP, vib. B3 level of theory (Debye) in Principal Axis frame

	$\mu_a e$	$\mu_b e$	$\mu_c e$	$\Delta\mu_a 0$	$\Delta\mu_b 0$	$\Delta\mu_c 0$
HETERO1	*1.6256*	*-0.9096*	*0.7876*	*-0.0534*	*0.0451*	*0.0093*
C1	*1.6314*	*-0.8955*	*0.7917*	*-0.0567*	*0.0597*	*0.0136*
C2	*1.6214*	*-0.9188*	*0.7856*	*-0.0513*	*0.0535*	*0.0096*
C3	*1.5990*	*-0.9563*	*0.7868*	*-0.0425*	*0.0603*	*0.0075*
C11	*1.6507*	*-0.8565*	*0.7948*	*-0.0507*	*0.0400*	*0.0039*
C12	*1.6396*	*-0.8884*	*0.7827*	*-0.0464*	*0.0437*	*0.0033*
C13	*1.6414*	*-0.8929*	*0.7738*	*-0.0398*	*0.0435*	*-0.0018*
C21	*1.6183*	*-0.9167*	*0.7944*	*-0.0473*	*0.0492*	*0.0026*
C22	*1.6167*	*-0.9262*	*0.7866*	*-0.0510*	*0.0560*	*0.0094*
C23	*1.5990*	*-0.9581*	*0.7846*	*-0.0540*	*0.0486*	*0.0050*

Table S31. The rPP equilibrium z-matrix coordinates and atom numbering of HOMO1.

NO	NA	NB	NC	Distance / Å	Angle / °	Dihedral angle / °	Mass / amu
1	0	0	0	0.000000	0.000000	0.000000	12.0000000
2	1	0	0	1.462885	0.000000	0.000000	12.0000000
3	2	1	0	1.500530	122.287673	0.000000	12.0000000
4	3	2	1	1.091884	110.508941	26.283267	1.0078250
5	3	2	1	1.093217	110.122844	-93.802616	1.0078250
6	3	2	1	1.091485	110.125900	146.869652	1.0078250
7	1	2	3	1.440712	59.520799	-103.394382	15.9949100
8	2	1	7	1.085384	116.655827	101.934878	1.0078250
9	1	7	2	1.086013	114.357494	-110.737098	1.0078250
10	1	7	2	1.084506	114.452476	110.710194	1.0078250
11	7	1	2	3.263424	95.318106	-102.276123	12.0000000
12	11	7	1	1.464503	87.058475	104.675200	12.0000000
13	12	11	7	1.501716	121.731546	177.634869	12.0000000
14	13	12	11	1.091303	110.528844	25.254806	1.0078250
15	13	12	11	1.093480	110.351278	-95.102825	1.0078250
16	13	12	11	1.092246	110.532774	145.432455	1.0078250
17	11	12	13	1.43510	59.43815	-103.62694	15.9949100
18	12	11	17	1.08714	117.24747	102.10142	1.0078250
19	11	12	13	1.08656	118.99600	-0.511400	1.0078250
20	11	12	13	1.08437	119.58586	153.754400	1.0078250
21	7	1	2	4.41770	145.94880	-26.08672	12.0000000
22	21	12	11	1.463822	70.777406	-31.926959	12.0000000
23	22	21	12	1.500155	122.154048	-168.243752	12.0000000
24	23	22	21	1.091666	110.449992	26.656441	1.0078250
25	23	22	21	1.093161	110.153992	-93.512120	1.0078250
26	23	22	21	1.090966	109.929003	147.224318	1.0078250
27	21	22	23	1.432910	59.551200	-103.944250	15.9949100
28	22	21	27	1.085940	117.381230	101.459020	1.0078250
29	21	22	23	1.086020	119.416680	-0.624980	1.0078250
30	21	22	23	1.086080	120.107460	152.920050	1.0078250

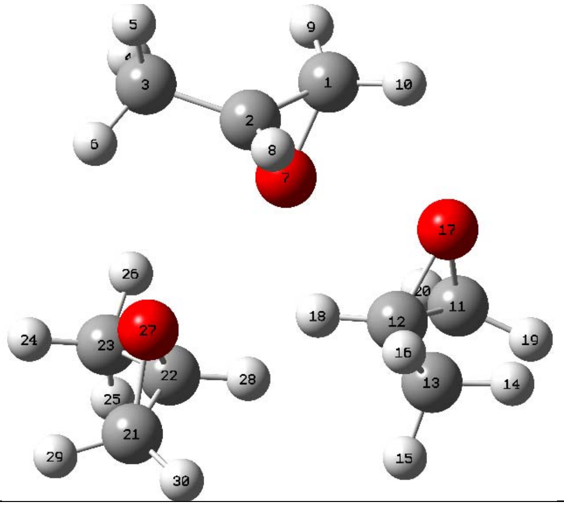

Table S32. The structural fitting results of HOMO1 based on B_0 and B_{semi}.

Parameters[a]	B_0 fit	B_{semi} fit	rPP cal. equil.
R(11, 7) /Å	3.3008 +/- 0.0021	3.2733 +/- 0.0094	3.2634
A(11, 7, 1) /°	96.088 +/- 0.045	96.10 +/- 0.23	95.318
R(21,7) /Å	4.37514 +/- 0.00076	4.3370 +/- 0.0031	4.4177
A(21,7,1) /°	147.736 +/- 0.014	147.080 +/- 0.065	145.949
Chi-squared	0.1513	3.0977	N/A
Deviation of fit	0.0763	0.3452	N/A

[a] All other structural parameters are fixed at the rPP values listed in Table S31 as well as atom numbers.

Table S33. The rPP equilibrium z-matrix coordinates and atom numbering of HETERO1.

NO	NA	NB	NC	Distance / Å	Angle / °	Dihedral Angle / °	Mass / amu
1	0	0	0	0.000000	0.000000	0.000000	12.0000000
2	1	0	0	1.462779	0.000000	0.000000	12.0000000
3	2	1	0	1.500538	122.127959	0.000000	12.0000000
4	3	2	1	1.091773	110.528249	27.093855	1.0078250
5	3	2	1	1.091711	110.052150	147.820236	1.0078250
6	3	2	1	1.093200	110.159230	-93.035716	1.0078250
7	2	1	3	1.085491	116.678665	-154.562317	1.0078250
8	1	2	3	1.440694	59.503452	-103.530859	15.9949100
9	1	8	2	1.084415	114.479452	110.621912	1.0078250
10	1	8	2	1.086052	114.359542	-110.740596	1.0078250
11	8	1	2	4.330035	146.774637	0.732136	12.0000000
12	11	8	1	1.464064	27.544373	166.753877	12.0000000
13	12	11	8	1.500345	122.064067	127.795203	12.0000000
14	13	12	11	1.093152	110.148467	93.629698	1.0078250
15	13	12	11	1.090741	109.891491	-146.928496	1.0078250
16	13	12	11	1.091874	110.439607	-26.499578	1.0078250
17	12	11	8	1.086424	117.636471	-77.422551	1.0078250
18	11	8	1	1.437588	35.496831	-50.464128	15.9949100
19	11	8	1	1.085469	111.475784	52.403375	1.0078250
20	11	8	1	1.085926	132.505664	-122.413554	1.0078250
21	8	1	2	3.276247	93.293655	-103.823231	12.0000000
22	21	8	1	1.463457	82.518018	111.276420	12.0000000
23	22	21	8	1.501269	121.774151	177.145897	12.0000000
24	21	8	1	1.438624	77.246917	50.925009	15.9949100
25	21	8	1	1.084758	43.055981	-98.794520	1.0078250
26	21	8	1	1.086284	158.321094	-74.422037	1.0078250
27	23	22	21	1.092218	110.480762	145.300586	1.0078250
28	23	22	21	1.093188	110.235438	-95.395109	1.0078250
29	23	22	21	1.091392	110.588069	25.068309	1.0078250
30	22	21	8	1.086056	117.278196	22.610211	1.0078250

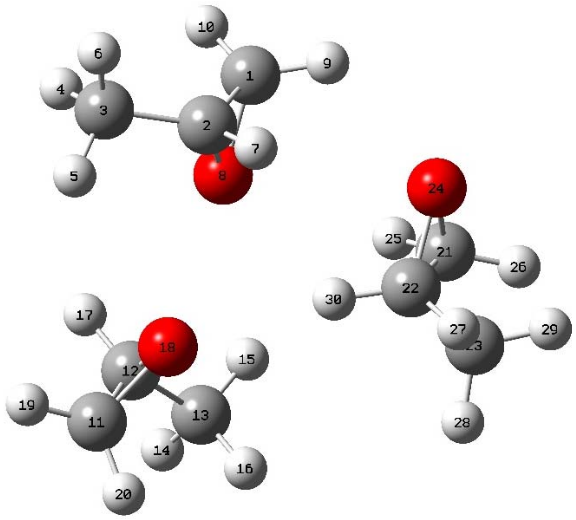

Table S34. The structural fitting results of HETERO1 based on B_0 and B_{semi}.

Parameters[a]	B_0 fit	B_{semi} fit	rPP cal. equil.
R(11, 8) /Å	4.3652 +/- 0.0011	4.3092 +/- 0.0065	4.3300
A(11, 8, 1) /°	147.392 +/- 0.014	146.781 +/- 0.080	146.775
R(21,8) /Å	3.3219 +/- 0.0014	3.2888 +- 0.0082	3.2762
A(21,8,1) /°	93.687 +- 0.022	93.54 +- 0.14	93.294
Chi-squared	0.1003	3.3869	N/A
Deviation of fit	0.0621	0.3609	N/A

[a] All other structural parameters are fixed at the rPP values listed in Table S33 as well as atom numbers.

Completion of reference 14.

Gaussian 16, Revision C.01. M. J. Frisch, G. W. Trucks, H. B. Schlegel, G. E. Scuseria, M. A. Robb, J. R. Cheeseman, G. Scalmani, V. Barone, G. A. Petersson, H. Nakatsuji, X. Li, M. Caricato, A. V. Marenich, J. Bloino, B. G. Janesko, R. Gomperts, B. Mennucci, H. P. Hratchian, J. V. Ortiz, A. F. Izmaylov, J. L. Sonnenberg, D. Williams-Young, F. Ding, F. Lipparini, F. Egidi, J. Goings, B. Peng, A. Petrone, T. Henderson, D. Ranasinghe, V. G. Zakrzewski, J. Gao, N. Rega, G. Zheng, W. Liang, M. Hada, M. Ehara, K. Toyota, R. Fukuda, J. Hasegawa, M. Ishida, T. Nakajima, Y. Honda, O. Kitao, H. Nakai, T. Vreven, K. Throssell, J. A. Montgomery, Jr., J. E. Peralta, F. Ogliaro, M. J. Bearpark, J. J. Heyd, E. N. Brothers, K. N. Kudin, V. N. Staroverov, T. A. Keith, R. Kobayashi, J. Normand, K. Raghavachari, A. P. Rendell, J. C. Burant, S. S. Iyengar, J. Tomasi, M. Cossi, J. M. Millam, M. Klene, C. Adamo, R. Cammi, J. W. Ochterski, R. L. Martin, K. Morokuma, O. Farkas, J. B. Foresman, and D. J. Fox, Gaussian, Inc., Wallingford CT, **2016**.
